# RNA binding protein Musashi1 interacts with the viral genomic RNA and restricts SARS-CoV-2 infection by repressing translation

**DOI:** 10.1093/nar/gkag271

**Published:** 2026-03-31

**Authors:** Sourav Ganguli, Divya Gupta, Rajashekar Varma Kadumuri, Dixit Tandel, Rajashree Ramaswamy, Aswathy G Krishnan, Deena T David, Soumya Bunk, Sreenivas Chavali, Krishnan Harinivas Harshan, Pavithra L Chavali

**Affiliations:** CSIR-Centre for Cellular and Molecular Biology, Hyderabad, Telangana 500007, India; Academy of Scientific and Innovative Research (AcSIR), Ghaziabad, Uttar Pradesh 201002, India; Department of Biology, Indian Institute of Science Education and Research (IISER) Tirupati, Tirupati, Andhra Pradesh 517619, India; CSIR-Centre for Cellular and Molecular Biology, Hyderabad, Telangana 500007, India; Department of Biology, Indian Institute of Science Education and Research (IISER) Tirupati, Tirupati, Andhra Pradesh 517619, India; CSIR-Centre for Cellular and Molecular Biology, Hyderabad, Telangana 500007, India; Academy of Scientific and Innovative Research (AcSIR), Ghaziabad, Uttar Pradesh 201002, India; CSIR-Centre for Cellular and Molecular Biology, Hyderabad, Telangana 500007, India; Department of Biology, Indian Institute of Science Education and Research (IISER) Tirupati, Tirupati, Andhra Pradesh 517619, India; CSIR-Centre for Cellular and Molecular Biology, Hyderabad, Telangana 500007, India; Academy of Scientific and Innovative Research (AcSIR), Ghaziabad, Uttar Pradesh 201002, India; CSIR-Centre for Cellular and Molecular Biology, Hyderabad, Telangana 500007, India; Department of Biology, Indian Institute of Science Education and Research (IISER) Tirupati, Tirupati, Andhra Pradesh 517619, India; CSIR-Centre for Cellular and Molecular Biology, Hyderabad, Telangana 500007, India; Academy of Scientific and Innovative Research (AcSIR), Ghaziabad, Uttar Pradesh 201002, India; CSIR-Centre for Cellular and Molecular Biology, Hyderabad, Telangana 500007, India; Academy of Scientific and Innovative Research (AcSIR), Ghaziabad, Uttar Pradesh 201002, India; Department of Biology, Indian Institute of Science Education and Research (IISER) Tirupati, Tirupati, Andhra Pradesh 517619, India

## Abstract

Musashi RNA-binding proteins are important post-transcriptional regulators of stem cell homeostasis and are known to be involved in viral infections. However, their role in SARS-CoV-2 infection remains largely unknown. Using computational studies, *in vivo* RNA immunoprecipitation, and biochemical assays, here, we establish that Musashi 1 (Msi1) interacts with viral genomic RNA through direct binding to the SARS-CoV-2 3′UTR. Importantly, binding of Msi1 to the viral 3′UTR results in translational repression that could be mediated by inhibition of poly(A) binding protein. Conversely, Msi1 knockout promotes robust viral replication and increased viral protein expression. Using 2D cell cultures, stem cells, and 3D organoids, we show that depletion of Msi1 in intestinal cells augments infection. This finding explains why the human intestine serves as a reservoir for SARS-CoV-2, in which differentiated enterocytes with negligible Msi1 levels are particularly affected. Contrarily, stem cells, which are enriched for Msi1 expression, are known to be less permissive to SARS-CoV-2 infection despite expressing the entry receptors. Our findings show how translational repression of SARS-CoV-2 by stem cell RNA-binding proteins, such as Msi1, could help evade infection.

## Graphical Abstract

**Graphical Abstract:**
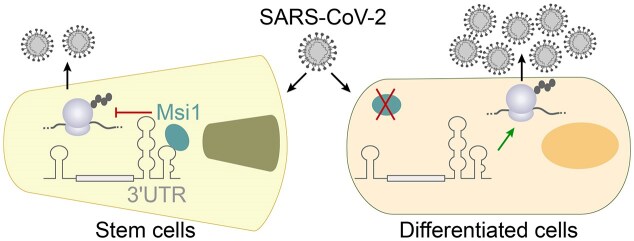

## Introduction

RNA viruses constitute the major fraction of the transmissible emerging pathogens, comprising ~223 geocoded viruses [1]. Since RNA viruses cause numerous infections and can lead to high mortality, understanding the molecular mechanisms underlying their pathogenesis is crucial for developing effective intervention strategies. In this context, the role of host RNA-binding proteins (RBPs), which interact with the viral RNA genome and modulate the viral life cycle, assumes significance in understanding viral biology and designing efficient inhibitors. Host RBPs are important for viral biology due to the dependence of the viral RNA genome on host cellular machinery for translation. Thus, the host RBPs predominantly affect the outcome of viral infection by affecting viral replication, stability, and translation [2]. For instance, genomes of many flaviviruses are enriched for Musashi binding elements (MBEs), which are recognized and bound by post-transcriptional regulators, Musashi 1 (Msi1) and Musashi 2 (Msi2) [3–5]. We previously showed that Msi1, a stem cell RBP, binds to the Zika RNA and promotes its replication by specifically binding to the 3′UTR of the viral genome [3]. Interestingly, overexpression of Msi1 in Zika non-permissive HEK293 cells promoted viral replication, demonstrating how RBPs could influence the cellular/tissue landscape of viral infection. Notably, RNA viruses are adept at molecular mimicry, exemplified by the rapid acquisition of RBP binding elements that sponge cellular RBPs, often resulting in beneficial outcomes for the viruses. For example, the more infective Brazilian strain of Zika had acquired an additional Msi binding element (MBE; GUAG) in its 3′UTR and showed an increased binding to the protein. This enables Zika viral RNAs to compete with the host RNAs for Msi1 binding, resulting in additional viral burden in the neural progenitor cells [3].

Conserved RNA-binding domains (RBDs) encoded within RBPs enable sequence-dependent recognition of an RNA. These RBDs typically recognize relatively short nucleotide sequences or preferentially bind to more simplified nucleotide patterns, such as purine-rich or pyrimidine-rich tracts of RNA. For instance, Msi1 and Msi2 bind RNA consensus sequences: 5′-[ GA]U_(1–3)_AGU-3′, through two conserved tandem RNA recognition motifs (RRMs; RRM1 and RRM2) [6]. In addition to the RRM sequences and cognate sites, the tissue context might also play a role in viral infection. For example, foetal neural progenitors are the prime targets of the Zika virus, while SARS-CoV-2 primarily infects differentiated cells [7–9]. SARS-CoV-2 infects the respiratory tract via angiotensin-converting enzyme 2 (ACE2) receptor attachment [10]. Once the virus enters pulmonary site by engaging its Spike protein with ACE2 or the transmembrane serine protease protein 2 (TMPRSS2), it can be either cleared from the mucociliary tract or enter gastrointestinal (GI) tract [11, 12]. The GI tract can serve as a reservoir for SARS-CoV-2, since the faecal samples of ~48%–54% of COVID-19 patients were positive for viral RNA, and ~15%–17% of patients exhibited gastrointestinal symptoms [13–15]. The virus can then trigger a cytokine storm, leading to moderate-to-severe pathologic manifestations [16].

The genomic RNA (gRNA) of SARS-CoV-2 serves as a bifunctional template, facilitating the synthesis of both full-length negative-sense RNAs required for genome replication and various sub-genomic negative-sense RNAs that are subsequently translated into corresponding sub-genomic messenger RNAs (mRNAs) [17]. Akin to other RNA viruses, SARS-CoV-2 depends on host proteins for the assembly of its replication and translation machinery [18]. Several studies have thoroughly characterized the SARS-CoV-2 RNA–protein interactome [19–22]. These studies show that the negative-sense RNAs are associated with an exceptionally diverse and extensive array of host proteins, which are involved in regulating viral production, apoptotic signalling, and immune responses. This underscores the critical role that negative-sense RNAs play during infection. For instance, the viral 5′UTR interacts with host proteins from the U1 small nuclear ribonucleoprotein (snRNP) family, while the viral 3′UTR interacts with host proteins associated with stress granules and heterogeneous nuclear ribonucleoprotein (hnRNP) families [23]. Notably, the conserved elements in 3′UTR of the SARS-CoV-2 comprise several domains that are important for controlling viral RNA production [24]. Here, we report that the SARS-CoV-2 genome contains conserved MBEs in its 3′UTR. We further demonstrate that Msi1 binds to the MBEs in the 3′UTR of viral genomic RNA, and this interaction limits the viral load by repressing viral translation.

## Materials and methods

### Estimation of binding-site opening energies based on single-strandedness

Following the method previously described [5], we employed a biophysical model that characterizes RNA at the secondary structure level, building upon the thermodynamic nearest neighbour energy model from the ViennaRNA Package [25]. This model facilitates the computation of the most stable, minimum free energy (MFE) structure of RNA and the partition function, 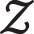. The equilibrium probability of a secondary structure is determined by the equation *p*(s)=(e −E(s)/RT)/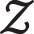. Efficiently computing the partition function, individual base pair probabilities can be determined for extensive sequences. The accessibility, or the probability that a segment along the RNA remains single-stranded, derives from this partition function. The accessibility of the model (probability of an RNA segment being single-stranded) is derived from this partition function. To do this, RNAplfold from the ViennaRNA package was used to compute the local pairing probabilities of GUAG, AUAG, and AGAA tetranucleotide motifs to gauge the single-strandedness.

We assessed the significance of the binding element opening energy by comparing it with a total of 1000 randomized sequences, maintaining the dinucleotide composition. Opening energies in both genomic and shuffled sequence contexts were compared using *z*-score statistics, defined as Z=E open(WXYZ)−μ/σ. Where E open(WXYZ) is the opening energy of tetranucleotide WXYZ in its genomic context, while μ and σ signify the mean and standard deviation, respectively, of the opening energies for WXYZ, determined from an extensive set of randomized sequences. Randomization involved dinucleotide shuffling of the 100-nucleotide windows upstream and downstream of the binding site, while maintaining the tetranucleotide motif sequence. An in-house Python script has been developed, encapsulating the methodology provided in the Perl utility plfoldz.pl, available at https://github.com/mtw/plfoldz. The tool utilizes ViennaRNA for thermodynamic calculations, reporting tetranucleotide opening energies alongside a *z*-score derived from 1000 dinucleotide shuffling events.

### Molecular dynamics simulations

All-atom molecular dynamics (MD) simulations were performed using the GROMACS 2019.3 simulation package [26, 27]. The Charm36 force field, tailored for the protein–RNA complex, was sourced from a prior study [28]. This complex was solvated in a dodecahedron box using the TIP3P water model, ensuring a minimum distance cut-off of 10 Å between the surface of the complex and the box edge. The system was neutralized with Na^+^ ions, and energy minimization was achieved using the steepest-descent algorithm until the energy converged to <1000 kJ/mol/nm. Before the production run, all systems were equilibrated for pressure and temperature by position-restraining the protein–RNA for 100 ps under both NPT (number of particles, pressure, temperature) and NVT (number of particles, volume, temperature) ensembles at designated temperatures. Long-range electrostatic interactions were computed using the particle mesh Ewald (PME) method [29] with a 0.16 nm grid spacing. A cut-off of 1.0 nm was designated for short-range electrostatic and van der Waals interactions. The LINCS algorithm [30] was used to constrain the bond lengths. All simulations were performed over a simulation timescale of 50 nanoseconds at a temperature of 300 Kelvin, using a 2-fs integration time step. A coupling coefficient of tT = 0.1 ps was employed, along with a modified Berendsen thermostat [31] and Parrinello–Rahman pressure coupling at 1 bar, both with a coupling coefficient of tP = 1 ps. Finally, data analysis was conducted using the Gromacs 2019.3 package.

### Modelling of RNA secondary structure and protein–RNA complex

The secondary structure of the reference viral 3′UTR sequence, possessing the lowest free energy, was predicted using the RNAstructure Fold webserver [32–34]. The analysis was conducted under default parameters at a temperature of 310.15 Kelvin. Final confirmation with a probability greater than 99% and a free energy of −74.7 kcal/mol was chosen for subsequent analysis. The two-dimensional representation of the predicted RNA structure was generated using the RNA2Drawer web server [35]. To investigate the binding efficiency of the Zika virus 3′UTR (WT) with the two MBEs in SARS-CoV-2, M1 (SL1) and M2 (HVR) were modelled along with that of the Zika virus 3′UTR (WT) using Discovery Studio Visualizer. Assisted docking of human Musashi 1 (PDBID: 5 × 3Z) with the designed RNA substrates was modelled in PyMOL, with inference from the solution structure of Msi1 RNA-binding domain 2 in complex with RNA (PDBID: 5 × 3Z).

### Constructs and primers

PUC57 vector containing SARS-CoV-2 minigene with Gaussia luciferase sequence was obtained from Dr Jingxin Wang [18]. From this, both the 5′UTR and the 3′UTR were polymerase chain reaction (PCR) amplified and cloned into the pJet1.2 blunt vector. To generate the M1 and M2 mutants, megaprimer-based mutagenesis [36] was performed using pJet1.2 3′UTR as the template with the following substitutions:

Stem loop 1 – GUAG >> GUGC at 113 nucleotide

Hypervariable Region 1 – GUAG >> GUGC at 292 nucleotide

Subsequently, double mutant was generated using a similar strategy, using M1 mutant as the template. Using this method, the SARS-CoV-2 minigene construct was mutated to generate a double mutant minigene construct for the Gaussia luciferase assay.

For the firefly luciferase assay, wild-type or the double-mutant 3′UTR from pJet 1.2 were cloned into pGL4.13 downstream of firefly luciferase by digestion with XbaI-HF (New England Biolabs). Short hairpin RNA (shRNA) targeting Msi1 and the scrambled shRNA were annealed using the established protocol and ligated to AgeI-HF (NEB) and EcoRI-HF (NEB) digested pLKO.1. DNA sequencing was used to confirm the sequence. Msi1 RRMs (Msi1:RRM; 1–190 residues in full-length Msi1) and Msi1 non-PABP binder (Msi1:ΔPBD, 190–235 residues in full-length Msi1) were amplified from the Msi1 wild-type construct and cloned into pEGFPC1. Primers used in this study are listed in Table 1.

**Table 1.** List of primers used in the study

Primer description	Sequence
Msi1 Forward	5′GTCGACTATGGAGACTGACGCGCCCCA3′
Msi1 Reverse	5′ATCGGATCCTCAGTGGTACCCATTGGTGA3′
SARS-CoV-2 3′UTR Forward	5′ACTCATGCAGACCACACAAGG3′
SARS-CoV-2 3′UTR Reverse	5′AAGAAGCTATTAAAATCACATGGG3′
SARS-CoV-2 5′UTR Forward	5′ATTAAAGGTTTATACCTTCCCAGG3′
SARS-CoV-2 5′UTR Reverse	5′CTTACCTTTCGGTCACACC 3′
PABP1 Forward	5′CACGTCGACATGAACCCCAGTGCCCCCA
PABP1 Reverse	5′ACAGGATCCTTAAACAGTTGGAACACCG 3′
Msi1 gRNA1	5′CCGCAGCAAGATGTTCATCG 3′
MSi1 gRNA2	5′GCTGTCGGTGAACACCACGG 3′
Scrambled shRNA	5′ACTAGTCGTTACATTAGTACTCAC 3′
Msi1 shRNA	5′ACGACGCCATGCTGATGTTTG 3′
SARS-CoV-2 3′UTR qPCR Forward	5′GAGGACTTGAAAGAGCCACCA 3′
SARS-CoV-2 3′UTR qPCR Reverse	5′AGAAGCTATTAAAATCACATGGGGA 3′
T7 M2 binding site	5′CAGTAATACGACTCACTATAGGGATTTTAGTAGTGCTATCCCCA3′
T7 M2 mutant binding site	5′CAGTAATACGACTCACTATAGGGATTTTAGCCGTGCTATCCCCA3′
T7 M1 binding site	5′CAGTAATACGACTCACTATAGGGTAGCACAAGTAGATGTAGTTAA3′
T7 M1 mutant binding site	5′CAGTAATACGACTCACTATAGGGTAGCACAAGCCGATGCCGTTAA3′
Msi1:ΔPBD Forward	5′CACCTCCTTTGGCTGAGCTTTCTTACAT3′
Msi1:ΔPBD Reverse	5′GCCCCTGGCTACACCTACCAGTTCCCCG3′
Msi1:RRM Reverse	5′ATCGGATCCCATCACCTCCTTTGGCTGAGCT3′

### Cloning and purification of recombinant Msi1 proteins

Human Msi1 was cloned into the PGEX 5X-2 vector using Not1-Sal1 enzyme. A single positive colony expressing GST–Msi1 was grown to mid-log phase (OD_600_ ≈ 0.6) and induced with IPTG (0.5 mM) and incubated for 4 h at 37°C. For His purification, Msi1 was cloned into pET28a(+) vector and induced with 1 mM IPTG at 18°C for 16 h. Cells were harvested by centrifugation, washed with STE buffer [10 mM Tris–HCl pH 8.0, 150 mM NaCl, 1 mM ethylenediaminetetraacetic acid (EDTA], and lysed in NETN buffer (20 mM Tris–HCl pH 8.0, 100 mM NaCl, 1 mM EDTA, 0.5% NP-40) supplemented with protease inhibitors, DTT, and Sarkosyl, followed by sonication. After removal of debris, the supernatant was incubated with Glutathione Sepharose beads/Ni-NTA column at 4°C to capture GST- or His-fusion proteins. Beads were washed with NETN buffer, and the bound proteins were eluted with glutathione buffer or increasing doses of imidazole. Protein expression and purity were confirmed using sodium dodecyl sulphate–polyacrylamide gel electrophoresis (SDS–PAGE).

### *In vitro* biotinylated RNA pulldown

Two micrograms of the wild-type and mutant SARS-CoV-2 5′UTR, 3′UTR, or an unrelated control RNA from the coding region of human WDR62 gene of the same length as SARS-CoV-2 3′UTR in pJet1.2 vector were linearized and subjected to *in vitro* transcription using Megashortscript T7 polymerase kit as per manufacturer’s instructions (Thermo Fisher Scientific) using Biotin-16 UTP (Cat#11388908910, Roche). The mixture was incubated for 3 h at 37°C, and EDTA (60 mM) was added and precipitated overnight at −20°C. After centrifugation at 14 000 × *g* for 30 min, the precipitate was dissolved in 50 μl of water and cleaned with Quick Spin clean-up columns (Roche). Following the DNase I treatment (Promega) for 15 min at 37°C, the reaction was cleaned once again with Bio-Spin 30 columns, and RNA was precipitated overnight at −20°C and dissolved in 30 μl of RNase-free water. Purity of RNA was analysed on agarose gel using RNA Gel Loading Dye (Thermo Fisher Scientific) after denaturation at 72°C for 10 min.

For the pulldown, three hundred micrograms of total protein from Caco-2 cells were used [lysis buffer: 25 mM Tris–HCl, pH 7.4, 150 mM KCl, 5 mM EDTA, 5 mM MgCl_2_, 1% NP40, 0.5 mM DTT, protease inhibitor cocktail (Roche), and 100 U/ml RNAse OUT] and first precleared with streptavidin beads (GenScript). The precleared lysate was diluted two-fold in lysis buffer and supplemented with tRNA (0.1 μg/μl, Thermo Fisher Scientific). It was then incubated with different concentrations of biotinylated RNA (5–20 pmol) for 2 h at 4°C. In the case of a pure protein pull-down, the appropriate amount of Msi1 protein was quantified and used. Biotinylated RNA was heated to 60°C for 5 min and then slowly cooled to room temperature. Subsequently, 50 μl of streptavidin beads were added for 1 h, and the beads were washed three times with lysis buffer containing 300 mM KCl. Finally, the beads were resuspended in 50 μl of sample buffer and boiled for 5 min at 95°C. After a short spin, the supernatant was collected and subjected to western blot analysis.

### RNA electrophoretic mobility shift assay

pJet 1.2 vectors containing full-length 3′UTR, 5′UTR, or the unrelated control RNA (from the coding region of the human WDR62 gene) were PCR amplified using T7 forward and pJet 1.2 reverse primers to generate templates. One microgram of purified template was *in vitro* transcribed using Megashort Script Kit (Thermo Fisher Scientific), incorporating Fluorescein-12UTP (Cat# 11427857910, Roche) in the reaction according to the manufacturer’s instructions. Likewise, the wild-type M1 and M2 and mutant constructs were annealed and subjected to an IVT reaction with Fluorescein-12UTP, and the reaction products were purified using a spin column (Roche) to remove unincorporated nucleotides. For the electrophoretic mobility shift assay (EMSA), 15 pmol of purified, labelled RNA was used as the template and incubated with the indicated amounts of GST–Msi1 in 1× EMSA buffer (20 mM Tris, pH 7.5, 150 mM NaCl, 0.5 mM EDTA, 0.1 μg/μl tRNA) for 45 min at 4°C. The GST corresponding to the highest GST–Msi1 concentration used in the experiment was employed as a control. Samples were resolved on gel and visualized using Cy2 laser in ImageQuant 800 (Cytiva).

### SARS-CoV-2 virus generation and infection

All viral experiments were carried out in the BSL-3 facility. Studies were performed using the B.1.1.8 variant of SARS-CoV-2, as previously described [37]. Viral transport medium with a CT value of <20 in real-time quantification was filter-sterilized (0.22 μm) and used as an inoculum for infecting Vero cells in a 96-well format. This cell culture supernatant was used to infect fresh Vero cells after the cytopathic effect was observed. This procedure was repeated until the supernatant showed an infectious titre of 10^−7^ ml^−1^. For variant identification and confirmation, the viral culture was sequenced using next-generation sequencing. Caco-2 cells (parental, Cas9 control, and Musashi1 knockouts) were infected with 1 multiplicity of infection (MOI = 1) in serum-free DMEM for 3 h at 37°C with 5% CO_2_. Following this, the cells were supplemented with 20% serum-containing DMEM. At 48 h post-infection (hpi), the supernatant was collected for real-time quantification and plaque assay, and the cells were harvested for western blotting.

### RNA immunoprecipitation assay

RNA immunoprecipitation (RIP) was performed from the lysates of the Caco-2 cells mock/infected with SARS-CoV-2 using rabbit IgG (Cat #12370, Sigma) and Msi1 (Cat #ab21628, Abcam) antibodies as previously reported [3]. Quality of immunoprecipitations was assessed by immunoblot analysis. At least 1.5 mg of protein lysate was made up to a volume of 500 μl and incubated with 8 μg of Musashi1 or IgG antibody for 3 h at 4°C, followed by the addition of 50 μl of prewashed protein A/G dynabeads (Life Technologies) for 2 h. Beads were then washed three times for 3 min each with lysis buffer, and RNA was isolated after the addition of 1 ml TRIzol (Life Technologies, cat. no. 15596-018). RNA isolated from 10% lysate was used as input. One-Step TB Green Mix (Takara) was then used for quantitative polymerase chain reaction (qPCR) analysis to evaluate the binding to 3′UTR and Meril One-Step qRT-PCR Kit (Thermo Fisher Scientific) using Nucleocapsid, Envelope, and RdRP as probes.

### UV cross-linking immunoprecipitation assay

UV cross-linking immunoprecipitation (CLIP) was performed as described previously [3] with minor modifications. Mock/infected Caco-2 cells were crosslinked with 300 mJ/cm^2^ of UVA (365 nm) on ice using the Stratalinker device. For the assay, the pellets were resuspended in 1 ml of NP40 lysis buffer and incubated for 15 min with constant mixing every 2 min. The lysates were then centrifuged at 10 000 × *g* for 15 min at 4°C. Subsequently, lysates were subjected to partial RNase If digestion (New England Biolabs, 5 μl 1:50 dilution per 1 ml of cell lysate) for 5 min at 37°C and verified on agarose gel. This lysate was incubated with Msi1-coated or IgG protein G Dynabeads (Life Technologies). Beads were then washed in NP40 lysis buffer, and DNaseI (20 U, Promega) was added for 15 min at 37°C following addition of 0.1% SDS and 0.5 mg/ml proteinase K (Thermo Fisher Scientific) for 15 min at 55°C. The supernatant was collected, and RNA was extracted using phenol/chloroform extraction and ethanol precipitation. Finally, RNA was dissolved in 20 μl of RNase-free water and qPCR was performed using One-Step TB Green Mix (Takara).

### SDS–PAGE and western blotting

Whole-cell lysates were loaded with equal amounts of protein and resolved on a 10% SDS–PAGE gel. The proteins were then transferred to a nitrocellulose membrane which was stained with Ponceau S and imaged. After imaging, the blot was incubated for 1 h in 5% non-fat lyophilized milk dissolved in 1× TBST, or in 5% BSA made in 1× TBST. It was then incubated overnight at 4°C or 2 h at room temperature with primary antibodies. The primary antibodies used are anti-Musashi1 (1:1000, Cat#ab21628, Abcam), anti-Musashi2 (1:1000, Cat#ab76148, Abcam), anti-p150Glued (1:2000, Cat#69399, Cell Signaling Technology), anti-GAPDH (1:5000, Cat# 2118; Cell Signaling Technology), anti-β-actin (1:10 000, Cat#3700, Cell Signaling Technology), anti-ACE2 (1:1000, Cat#92485, Cell Signaling Technology), anti-Nucleocapsid (1:8000 in 5% BSA, Cat#MA5-29981, Invitrogen ), anti-GST (1:5000, Cat#2625, Cell Signaling Technology), anti-His (1:2000, Cat#2365, Cell Signaling Technology), and anti-GFP (1:2000, Cat# 2555; Cell Signaling Technology). After overnight incubation, the blot was washed three times for 5 min each with 1× TBST to remove any non-specific binding of the primary antibody. The blot was then incubated for 1 h at room temperature with the appropriate secondary antibody. The secondary antibodies commonly used in our study were anti-mouse HRP-conjugated secondary (1:4000; Cat# ab97023, Abcam) and anti-rabbit secondary IgG (1:5000, Cat# NB7156, Novus Biologicals). After incubation the blot was again subjected to three washes and then developed using HRP-luminol-based chemiluminescence (Clarity Max ECL, Biorad) in Vilber-Lourmat ChemiDoc machine.

### Real-time quantification

RNA from cell culture supernatant was isolated using viral RNA isolation kit (MACHEREY-NAGEL GmbH & Co. KG). Real-time quantitative PCR (RT-PCR) was performed in Roche LightCycler 480 using commercial kits. LabGun™ COVID-19 RT-PCR kit was used to measure the RNA levels of SARS-CoV-2 RdRp and E gene levels, following manufacturers’ protocol. Fold changes between samples were calculated by ΔΔCp method using the internal control (IC) for normalization. The RNA was diluted with nuclease-free water in a ratio of 1:40 and 1 μl was used for a 10 μl reaction with One Step CT Power SyBR Green Mix (Thermo Fisher Scientific) or TB Green Mix (Takara) as per the manufacturers’ instructions.

### Lentiviral preparation and genomic modifications of cell lines

Cas9 lentivirus was generated by transfecting 10 μg of Lenti-Cas9-Blasticidin plasmid (Transomic Technologies) in HEK293T cells along with 2.5 μg of psPAX-2 and 7.5 μg of pMD2. Caco-2 cells stably expressing Cas9 were generated after 10 μg/ml of blasticidin selection for a week. Msi1 knockouts were then generated in this background (parental) using transEDIT-dual CRISPR gRNA lentiviral expression vector (TEDH-1049415) with target sequences of 5′CCGCAGCAAGATGTTCATCG3′ and 5′GCTGTCGGTGAACACCACGG3′. These cells were selected with puromycin (1.5 μg/ml for 96 h) to generate Musashi1 knockout pool. Subsequently, they were diluted in a 96-well plate for clonal expansion of single genotypes and screened using western blotting to confirm the absence of Musashi1. Transient rescues were made using Lipofectamine 3000 (Thermo Fisher Scientific)-based expression of transgene Msi1 tagged with GFP.

### Cell and organoid culture

Caco-2 cells were maintained in DMEM GlutaMax (Gibco), supplemented with 20% (v/v) foetal bovine serum (FBS—Gibco). HEK293T and HEK293 cells were maintained in DMEM GlutaMax (Gibco), supplemented with 10% (v/v) foetal bovine serum (FBS—Gibco). Induced pluripotent stem cells were purchased from Thermo Fisher Scientific and maintained in mTeSR1 media (Cat#85850, Stem Cell Technologies Inc). All cells were grown and incubated at 37°C and 5% CO_2_ and tested monthly for mycoplasma using a PCR-based assay. Intestinal organoids were generated using STEMdiff intestinal organoid kit (Cat#05140, Stem Cell Technologies Inc), and endoderm identity was confirmed with SOX17 staining.

### Luciferase assay

*In vitro* transcription was carried out to amplify wild-type or the double mutant 3′UTR luciferase from pGL4.13-constructs. HEK293T cells were plated in a 48-well plate at 60% confluency and transfected with the empty pEGFP-C1 plasmid, pEGFP-C1-Msi1, pEGFP-C1-Msi1ΔRRM, pEGFP-C1-Msi1ΔPBD, and pRL-SV40. After 24 h, cells were transfected with 200 ng of the wild-type or MBE double-mutant luciferase RNA construct. After 24 h, cells were lysed, and luciferase assay was performed using the Dual Luciferase Reporter Assay System (Cat# E1910, Promega). The data were obtained using a PerkinElmer microplate reader, and the firefly-to-renilla luciferase ratio was estimated.

For the Gaussia luciferase assay, HEK293T cells were plated in 24-well plate at 70% confluency and transfected with the empty pEGFP-C1 plasmid and the pEGFP-C1-Msi1 plasmid. After 24 h, equal amounts (145 ng) of capped wild-type or mutant minigene RNA were transfected. Minigene RNA was generated using M13 forward and reverse PCR-amplified fragment using mMessage mMachine T7 transcription kit as per manufacturer’s instructions. After 48 h of minigene RNA transfection, cell culture media was collected and centrifuged at 1500 × *g* for 5 min to remove cell debris. Twenty microlitres of the media was subjected to Luciferase assay using Pierce Gaussia glow assay kit (Cat# 16161, Thermo Fisher Scientific).

### Statistical analysis

Two-tailed t-tests were used to determine statistical significance of differences in distribution across all experiments. *P*-values for each comparison are indicated in graphs. Each experiment was performed using a minimum of three biological replicates. All viral infections and qPCR experiments were performed double-blind.

## Results

### The 3′UTR of SARS-CoV-2 genomic RNA contains conserved MBEs

**Figure 1.**
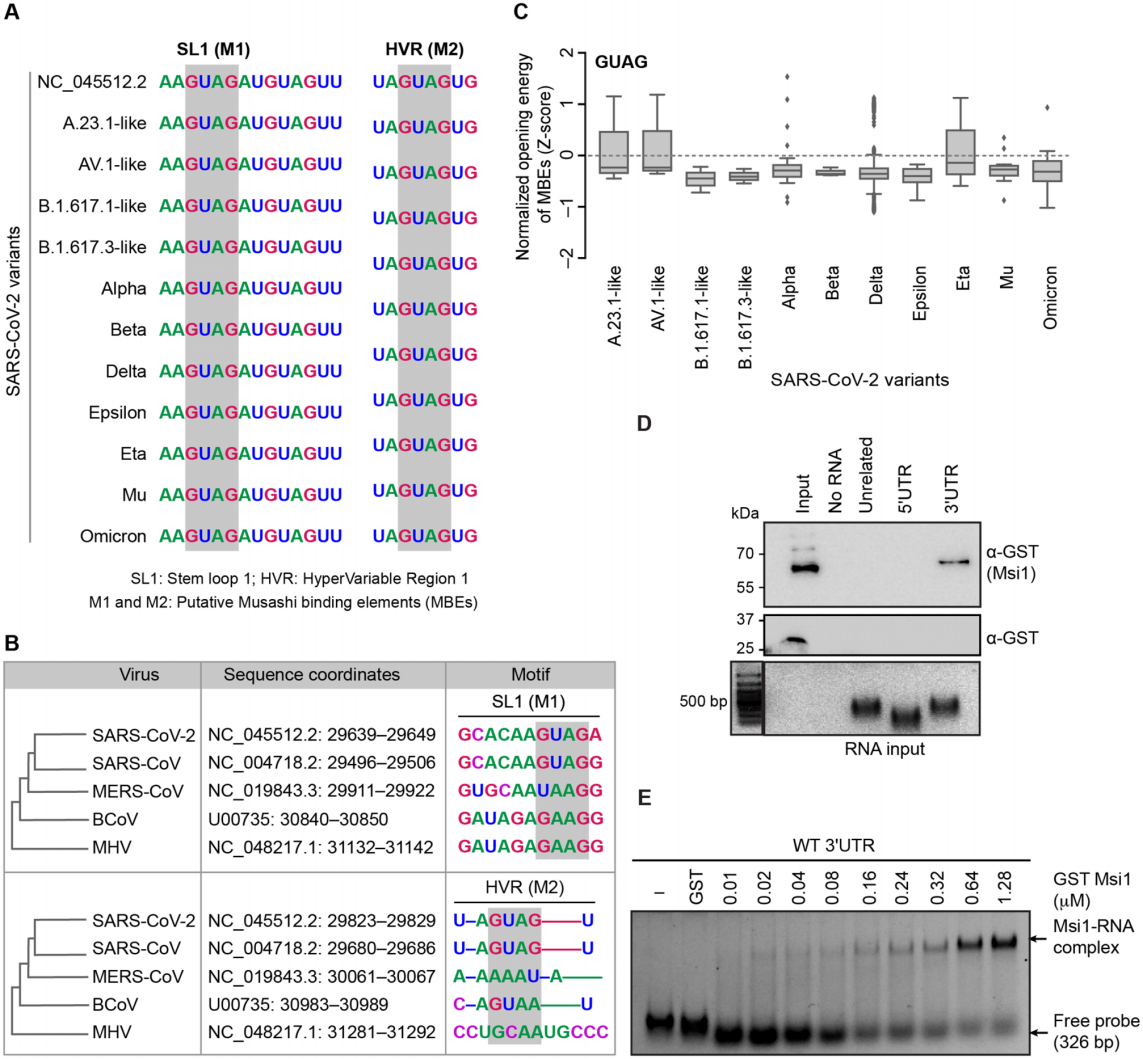
SARS-CoV-2 3′UTR contains conserved MBEs. (**A**) MBEs from different variants of SARS-CoV-2 highlighting their conservation status. (**B**) Multiple sequence alignment of MBEs in coronaviruses.(**C**) Box plot of distribution of normalized opening energies for (GUAG) Msi consensus site across SARS-CoV-2 variants. (**D**) RNA pull-down assays were performed using recombinant GST–Msi1 (top panel) or GST alone (bottom panel) incubated with *in vitro*–transcribed SARS-CoV-2 3′UTR RNA, followed by immunoblot detection with an anti-GST antibody (α-GST). The SARS-CoV-2 5′UTR and an unrelated RNA of the same length (326 bp) as the SARS-CoV-2 3′UTR were used as controls. RNA input levels are shown. (**E**) RNA EMSA assay showing the binding of GST–Msi1 (ranging from ∼ 0.01 to 1.28 μM) to full-length labelled 3′UTR in a dose-dependent manner as indicated. The highest GST concentration was used as the control. Arrow marks indicate the free unbound probe and the Msi1–RNA complex.

Sequence analysis of the 5′UTR and 3′UTR across 224 variants of SARS-CoV-2 revealed the presence of two putative conserved MBEs, defined by GU_(1–3)_AG, specifically in the 3′UTR (Fig. 1A). The two putative MBEs (henceforth referred to as M1 and M2) were located in (i) conserved loop 1 of Stem and Loop 1 structure (SL1; M1) and (ii) the more divergent hypervariable region (HVR; M2) (Supplementary Fig. S1A). The SL1 structure is essential for viral replicability, while the HVR region may contribute to viral pathogenicity [38]. Notably, both the MBEs are conserved in SARS-CoV and SARS-CoV-2, but not in Middle East respiratory syndrome (MERS), Bat or Mouse Hepatitis Virus (MHV) viruses (Fig. 1B). We next estimated the free energy required to force the MBE to be single-stranded (opening energy) using a previously described method [5]. Strikingly, across all variants, including Omicron, we identified that GUAG in the MBEs exhibited low opening energies with *z*-scores ranging from −1.10 to 1.53, reflecting their accessibility in the genome throughout the evolution of SARS-CoV-2 (Fig. 1C and Supplementary Fig. S1B). Contrarily, both the AUAG and the recently shown AGAA motifs, which have the propensity to bind to Msi1 [6], showed a higher median distribution of binding energies, alluding to a possible unfavourable context for Msi1 binding (Supplementary Fig. S1C). To check if Msi1 binds directly to the 3′UTR of SARS-CoV-2, we performed RNA pulldown assay with GST-tagged Msi1 and the *in vitro* biotinylated SARS-CoV-2 3′UTR, 5′UTR, and an unrelated RNA control. We identified that Msi1 binds specifically to SARS-CoV-2 3′UTR, while we did not detect any binding to the unrelated RNA or the 5′UTR of SARS-CoV-2 (Fig. 1D and Supplementary Fig. S2A). We further confirmed direct binding to the 3′UTR by employing RNA EMSA, which showed a dose-dependent increase in Msi1-3′UTR RNA complex formation (Fig. 1E and Supplementary Fig. S2B).This was also verified using His-tagged Msi1, which bound at a concentration as low as 5 pmol of RNA and saturated at 10 pmol of RNA with 60 nM of Msi1 protein (Supplementary Fig. S2C). 

### Msi1 binds to conserved MBEs in 3′UTR of SARS-CoV-2 *in vitro* and in cells

**Figure 2.**
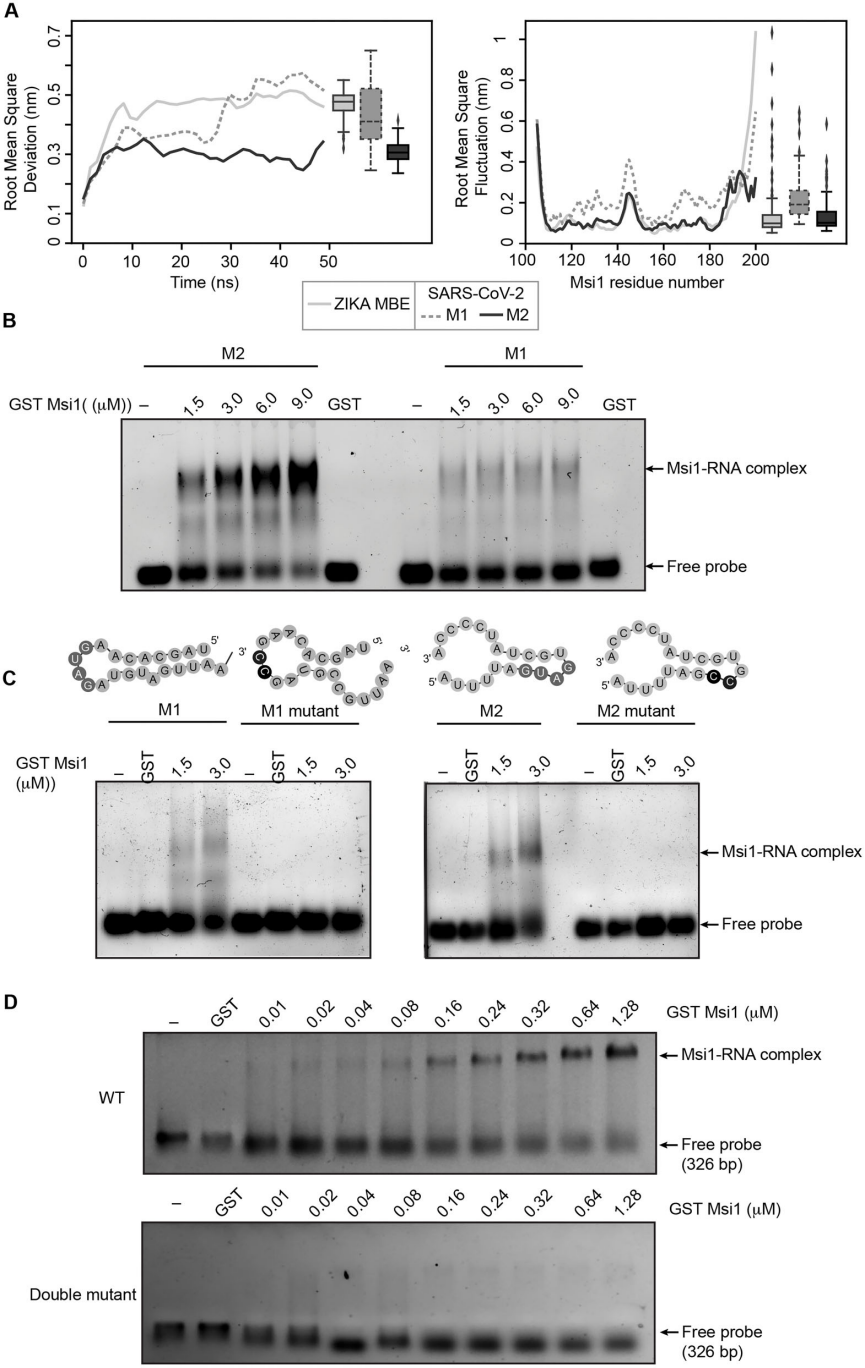
Msi1 directly binds to SARS-CoV-2 3′UTR. (**A**) Comparison of MD simulations of Zika virus 3′UTR (WT) with the two MBEs in SARS-CoV-2, M1 (SL1) and M2 (HVR1). (**B**) RNA EMSA depicting dose-dependent binding of GST–Msi1 to the *in vitro* transcribed MBEs—M1 and M2. We incubated 15 pmol of M1 and M2 probes with increasing concentrations of GST–Msi1 (∼1.5, 3, 6, and 9 μM) or the highest concentration of GST alone. Arrows indicate free unbound probe and the Msi1–RNA complex. (**C**) RNA EMSA showing the specificity of Msi1 binding to the MBEs in SARS-CoV-2 3′UTR. Images show dose-dependent binding of GST–Msi1 to the *in vitro* transcribed MBEs (M1, M2) and their respective mutants. MBEs are indicated using dark grey circles and mutants in black-shaded circles. (**D**) RNA EMSA depicting the loss of Msi1 binding to the full-length SARS-CoV-2 3′UTR upon mutations in both M1 and M2 binding sites (double mutant).

To assess the preference of human Msi1 binding to the two MBEs in the SARS-CoV-2 3′UTR, we next performed MD simulations of the two sites (M1 and M2) and compared them with the Zika 3′UTR. The variation in the RMSD and RMSF of Msi1 in complex with M2 in the HVR was less in comparison to Zika 3′UTR than that of Msi1 in complex with M1 (Fig. 2A and Supplementary Fig. S3A). To validate this interaction *in vitro*, we performed RNA EMSA using *in vitro*–transcribed RNAs corresponding to the M1 and M2 sequences. While RNA–Msi1 complexes were detectable at both sites, binding to the M2 RNA was robust and showed a clear dose-dependent increase with increasing Msi1 concentrations. In contrast, binding to the M1 RNA was weak and non-dose-responsive under the same conditions, suggesting that efficient Msi1 association at this site may require additional cofactors or higher-order RNA structural context not captured in the *in vitro* EMSA assay (Fig. 2B). Importantly, mutation of the predicted Msi1 binding sites in both M1 and M2 abolished complex formation, confirming the specificity of Msi1–RNA interactions, as the mutant RNAs failed to bind Msi1 under identical conditions (Fig. 2C and Supplementary Fig. S3B). Taken together, our results suggest that the M2 site exhibits robust and dose-dependent complex formation, demonstrating it as the primary Msi1 binding site. We performed an RNA EMSA experiment using double mutant in the full-length 3′UTR and observed a lack of complex upon loss of both the binding sites (Fig. 2D). These results were also recapitulated in the RNA pull down experiments (Supplementary Fig. S3C).

**Figure 3.**
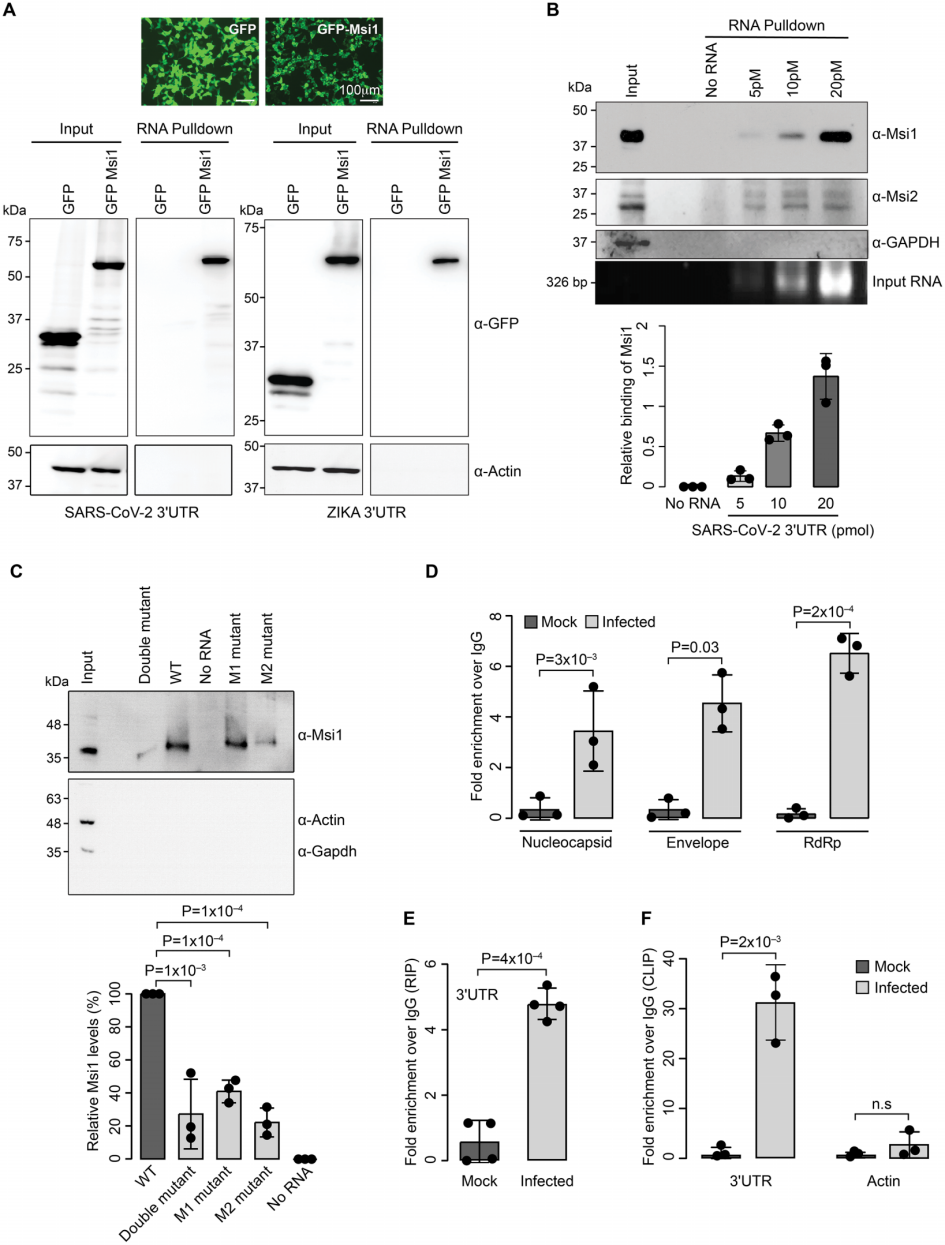
Msi1 binds to SARS-CoV-2 genomic RNA in cells. (**A**) RNA pull-down assays performed with the 3′UTRs of SARS-CoV-2 and Zika virus. *In vitro* transcribed biotinylated 3′UTR RNA from SARS-CoV-2 and Zika virus was incubated with GFP or GFP–Msi1-expressing HEK293 cell extracts, and RNA–protein complexes were captured on streptavidin beads. Representative image of western blots probed with antibodies against GFP–Msi1 and Actin as loading control. The panel on top depicts the transfection efficiency of GFP and GFP–Msi1. (**B**) RNA pull-down assays were performed with increasing concentrations of the 3′UTR of SARS-CoV-2. Different concentrations of biotinylated RNA were incubated with Caco-2 cell extracts and RNA–protein complexes were captured on streptavidin beads. Representative image of western blots probed with antibodies against Musashi-1 (Msi1), Musashi-2 (Msi2), and GAPDH as a control. Corresponding protein and RNA inputs are shown. The graph at the bottom shows a densitometric analysis of Msi1 binding to increasing concentrations of SARS-CoV-2 3′UTR. (**C**) RNA pull-down assays of endogenous Msi1 from Caco-2 lysate with the wild-type 3′UTR, M1, M2, and double mutant of SARS-CoV-2 3′UTR. Representative image of immunoblots probed with antibodies against Msi1 and with Actin and GAPDH as a loading control. Graph on the right provides the densitometric analysis depicting the amount of Msi1 in RNA pull-down for the corresponding 3′UTR of SARS-CoV-2. (**D**) Msi1 RIP from mock- and SARS-CoV-2-infected Caco-2 cells. The graphs depict the Msi1-bound viral genomic RNA using Nucleocapsid, Envelope, RdRp probes, and (**E**) 3′UTR. Rabbit IgG or Msi1 antibodies were used for immunoprecipitation. Nucleocapsid and Envelope transcripts were quantified by TaqMan assay, whereas 3′UTR was quantified with SYBR qPCR. RIP values are presented as fold enrichment over the IgG. Bar charts depict mean ± SD. *n* = 3 biological replicates for TaqMan assays and *n* = 4 for SYBR-based assays. *P*-values were obtained using a two-tailed paired Student’s t-test. (**F**) Msi1 CLIP from Caco-2 cells with mock and infected cells as indicated. CLIP was performed with rabbit IgG or Msi1 antibodies. Graphs show qPCRs of bound transcripts, measured across three biological replicates. Specificity of the Msi1 CLIP was verified by performing qPCR of Actin transcript. *P*-values were obtained using two-tailed paired Student’s *t*-test.

To examine if Msi1 binds to the SARS-CoV-2 3′UTR in cells, we transfected GFP-tagged Msi1 into HEK293 cells and performed *in vitro* RNA pull-down with biotinylated SARS-CoV-2 3′UTR, using Zika 3′UTR as a positive control. Indeed, Msi1 showed binding to both the SARS-CoV-2 3′UTR and the Zika 3′UTR (Fig. 3A). To confirm that the endogenous Msi1 could bind to the 3′UTR of SARS-CoV-2, we performed RNA pull-down with the colon carcinoma cell line (Caco-2), which expresses endogenous Msi proteins. We find that both Msi1 and Msi2 can bind to the SARS-CoV-2 3′UTR (Fig. 3B). These findings suggest that the 3′UTR of SARS-CoV-2 contains well-conserved Musashi binding sites and is amenable to binding to Msi proteins in a cellular context.

To consolidate the findings from RNA EMSAs, we performed RNA pull-down assays using M1, M2, and the double mutant constructs expressed in Caco-2 cells. In agreement with the EMSA data, we find that the mutation of the M1 site alone did not perturb Msi1 binding, and showed that the M2 mutant as well as the double mutant (M1 and M2) showed almost complete abrogation of binding, indicating that both these sites could contribute to binding, while the M2 site might be the primary contributor to Msi1 binding to SARS-CoV-2 3′UTR (Fig. 3C). To examine whether Msi1 binds to the SARS-CoV-2 3′UTR in cells during viral infection, we performed RIP in Caco-2 cell lines infected with SARS-CoV-2. We identified that Msi1 effectively precipitated SARS-CoV-2 RNA from infected cells, as indicated by the amplification of viral Nucleocapsid, Envelope, and RdRp transcripts, as well as the viral 3′UTR (Figs. 3D and E). Moreover, UV crosslinking followed by RIP (CLIP) revealed a robust direct interaction of Msi1 with the 3′UTR of the SARS-CoV-2 genomic RNA (Fig. 3F). Collectively, these findings establish that Msi1 binds to the SARS-CoV-2 3′UTR at the two MBEs present in the SL1 and HVR.

### Depletion of Msi1 levels increases viral load and infectivity

**Figure 4.**
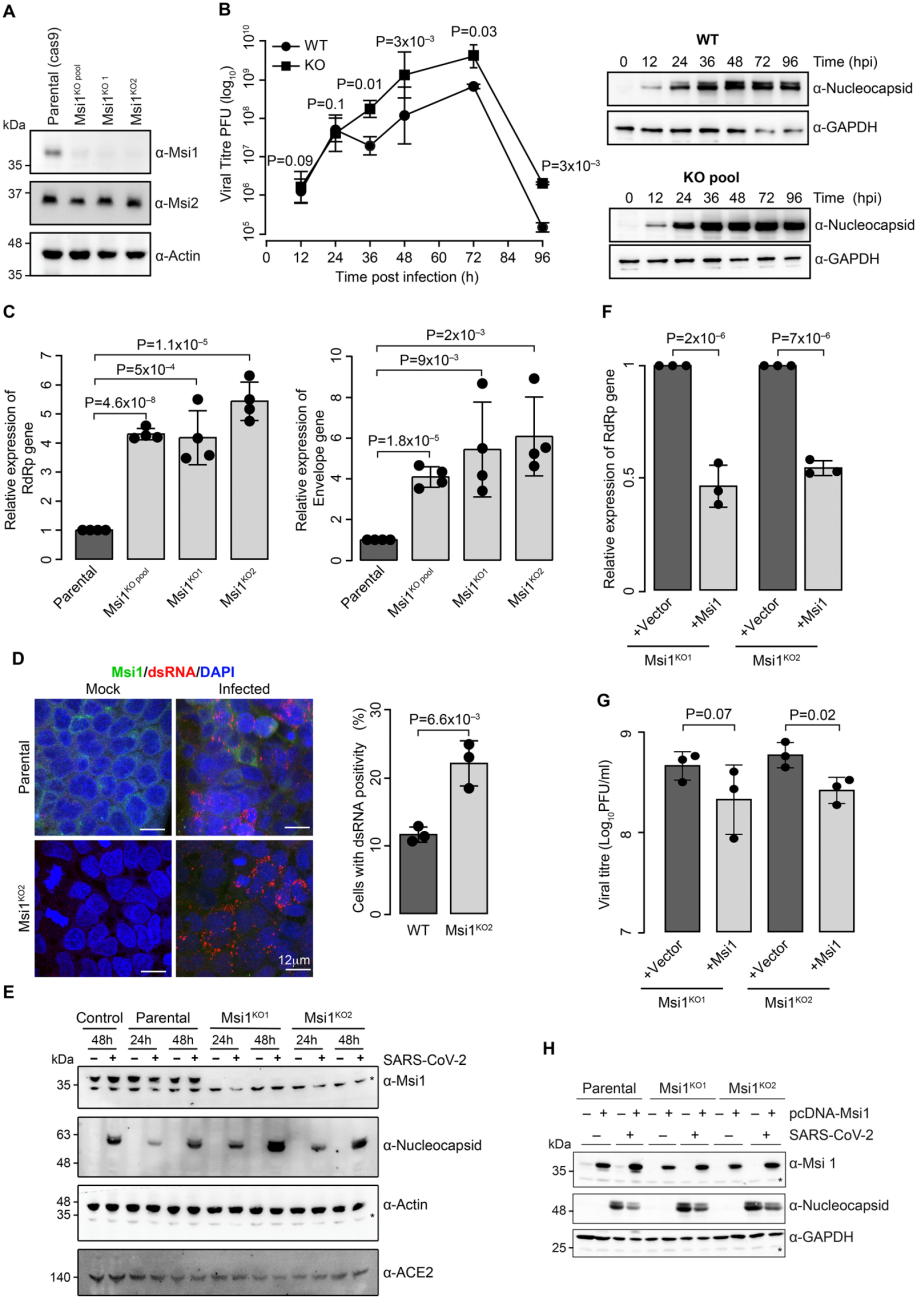
Msi1 knockout enhances SARS-CoV-2 infectivity in cells. (**A**) Western blot analysis of Caco-2 knockouts (KO) depicting the absence of Msi1 protein and unaltered levels of Msi2. Actin was used as a loading control. (**B**) Line graph showing growth kinetics of SARS-CoV-2 in control versus Msi1 KO cells over indicated time points measured by plaque formation assay. Data from three independent experiments were plotted and *P*-values indicate significance in the difference of the distributions at each time point. The panel on the right shows nucleocapsid protein assayed by western blot analysis from the matched infected samples used in plaque formation assay. (**C**) The graph showing viral RNA copies in Msi1 KO pool or individual KO clones 1 and 2 following infection with SARS-CoV-2. All experiments were done at MOI = 1 PFU/cell after 48 h. In all viral replication assays, RdRp and Envelope transcripts were quantified using a TaqMan assay. *P*-values for *n* = 4 biological replicates were estimated using two-tailed paired Student’s t-test. (**D**) Confocal microscopy images of SARS-CoV-2-infected parental and KO2 Caco-2 cells immunostained with antibodies against dsRNA (red), Msi1 (green), and DAPI (blue) following mock or SARS-CoV-2 infection. (**E**) Western blots of mock- or SARS-CoV-2-infected control (WT), parental (Cas9), and Msi1 KO cell lines with indicated genotypes across the indicated time points. Blots were probed with indicated antibodies. Actin was used as a loading control. *indicates non-specific band. (**F**) Viral transcript measured using the RNA levels of RdRp gene in Msi1 KO cells rescued with empty vector or Msi1 transgene following infection with SARS-CoV-2. *n* = 3 biological replicates. (**G**) Quantitation of the infectious viral titres of SARS-CoV-2 in Msi1 KO cells rescued with Vector or Msi1. (**H**) Western blot analysis of cell lines with indicated genotypes, mock-infected or infected with SARS-CoV-2 (MOI = 1, 48 h). Blots were probed with antibodies as indicated, with GAPDH serving as a loading control.

To investigate the physiological relevance of Msi1 interaction with SARS-CoV-2 3′UTR, we generated Msi1 knockouts (KO) in Caco-2 cells using lentivirus-based CRISPR–Cas9. Importantly, the protein levels of Msi1 remain unchanged at different time points following SARS-CoV-2 infection in Caco-2 cells (Supplementary Fig. S4A). Therefore, we generated Msi1 KO cells and verified the protein levels of both Musashi proteins (Msi1 and Msi2) in the KO pools, as well as in individual knockouts. The guides specifically targeted Msi1, and we could not detect any change in Msi2 protein levels upon loss of Msi1 (Fig. 4A). We then subjected the parental cells expressing Cas9 and Msi1 knockout cells to infection with SARS-CoV-2. Viral growth kinetics post-infection were assessed using the plaque assay with supernatant collected at different time points after infection. While there were no significant changes in infectivity at 12 h and 24 h, we observed a significant increase in infectivity in the Msi1-KOs from the 36 h time point, which persisted until 96 h (∼8–11 folds). This was also reflected in the alterations in the viral protein levels (Fig. 4B). We also observed a consistent alteration in plaque formation in the individual Msi1 KO clones (Supplementary Fig. S4B). Since we observed a maximum differential response at 36–48 h post-viral infection, subsequent studies were performed at 48 h post-infection. Viral transcript analysis post-infection revealed a significant increase in Envelope and RdRp transcript levels across the Msi1 KO pool and individual clones, suggesting increased viral replication (Fig. 4C). This increase in viral replication was supported by greater double-stranded RNA (dsRNA) positivity in Msi1 KO cells than in control cells (Fig. 4D). We then performed western blot analysis to compare nucleocapsid levels between control and knockout cells at 24 h and 48 h post-infection. We observed a ~2–3-fold increase in nucleocapsid protein levels in the Msi1 knockouts starting from the 24-h time point, peaking at the 48-h time point to ~4-fold (Fig. 4E). Given that Msi1 KO cells exhibited a significant increase in viral titres, we reintroduced the Msi1 transgene into the knockout background to assess whether the inhibitory effect of Msi1 could be restored in the KO cell lines (Fig. 4F). Upon re-expression of Msi1 in the KO background, we observed a 50% reduction in the expression levels of the viral RdRp transcripts when compared to the parental KOs (Fig. 4F). This result was corroborated by a plaque assay, in which the reintroduction of Msi1 showed a reduction in plaque formation compared to the parental knockouts (Fig. 4G and Supplementary Fig. S4C). Although the rescue did not phenocopy the parental cells, it was significantly different from the Msi1 KOs. The differences between the rescue and the parental cells could be attributed to the clonality and transfection efficiency of Caco-2 cells. Immunoblot analysis of Msi1 KO cells with transgene expression and subsequent infection demonstrated decreased levels of viral nucleocapsid protein, confirming the inhibitory role of Msi1 in viral replication (Fig. 4H).

### Msi1 depletion in the stem cells promotes viral infection

Msi1 is known to affect intestinal homeostasis by controlling stem cell proliferation [39, 40]. The gut is also recognized as a reservoir for SARS-CoV-2 [41, 42]. While Caco-2 cells enable the controlled analysis of viral infection in colonocyte- and enterocyte-like cells [43], stem cells and organoids would better capture the Msi1-rich stem cell niche and the native cellular diversity of the human gut. Thus, we investigated the role of Msi1 in regulating SARS-CoV-2 infection in stem cells and organoids. The ileum and colon are known to exhibit elevated expression of the entry receptors for viruses, specifically ACE2, making them more susceptible to infection [44, 45]. To assess whether Msi1 affects ACE2 levels, we performed an immunoblot assay and found that there was no change in ACE2 expression in Msi1 KOs Caco-2 cells following infection (Fig. 4E). This suggests that Msi1 might not regulate the viral entry by modulating ACE2 levels.

**Figure 5.**
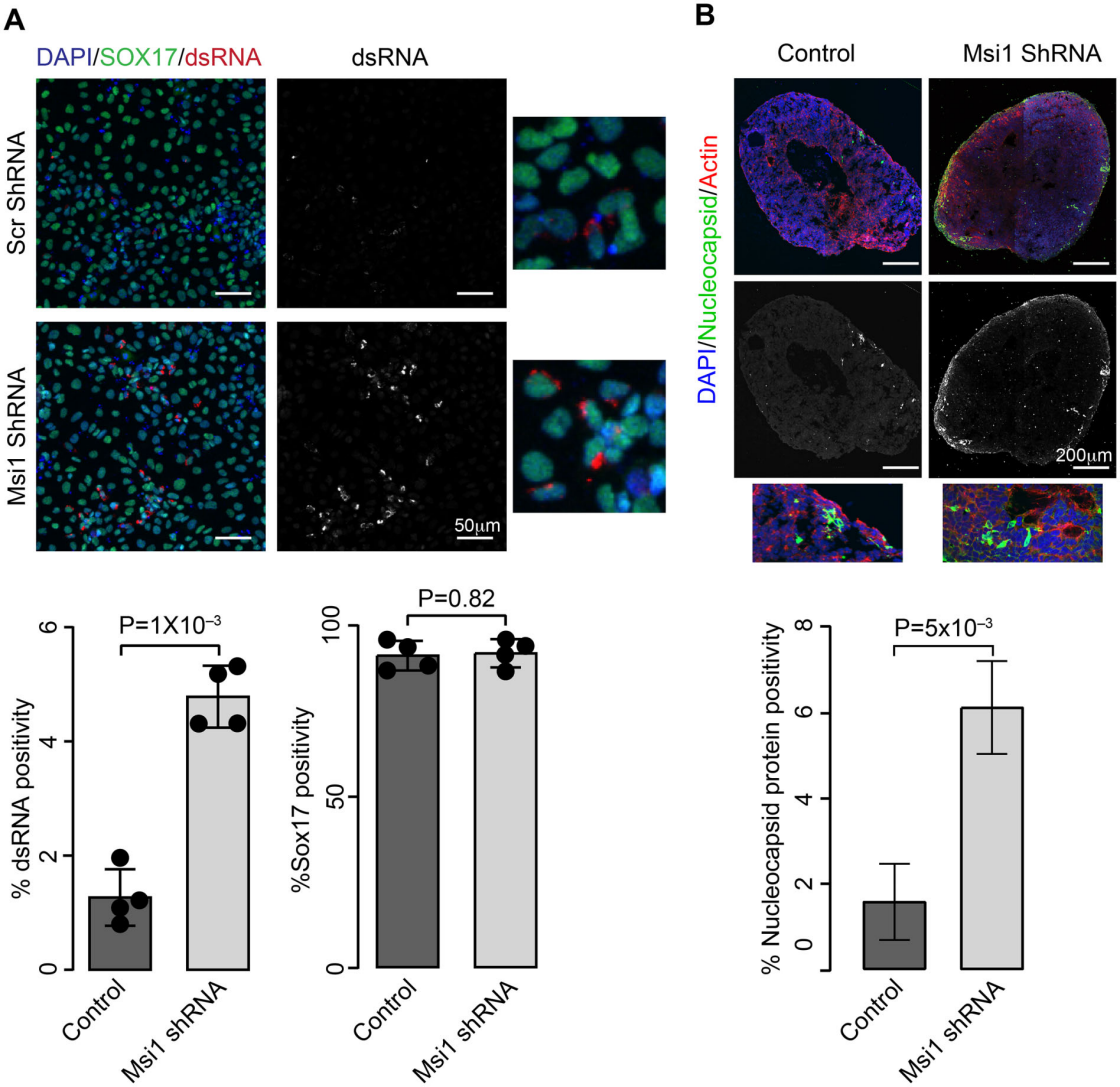
Msi1 depletion enhances SARS-CoV-2 infection in DE cells and gut organoids. (**A**) Confocal images of DE cells treated with scrambled- or Msi1-shRNA subjected to SARS-CoV-2 infection (MOI = 1, 48 hpi). SOX17 depicts DE cells, dsRNA represents viral replication, and DAPI the nuclei. The graphs depict quantification of dsRNA and SOX17 positivity (%) in each condition. (**B**) Confocal microscopy images of infected gut organoids treated with scrambled shRNA or Msi1 shRNA immunostained with antibodies against Nucleocapsid protein (green), Actin (red), and DAPI (blue). The graph depicts quantification of Nucleocapsid protein positivity normalized to DAPI.

Previous studies have demonstrated that intestinal organoids are susceptible to SARS-CoV-2 infection, with enterocytes serving as the primary targets, resulting in the production of infectious viral particles and epithelial damage [7, 46]. The Msi family of proteins plays a critical role in maintaining intestinal regeneration and homeostasis by promoting the exit of intestinal stem cells from quiescence to an active proliferative state, especially after injury to the intestinal epithelium [47]. In this context, here, we first examined the effect of Msi1 depletion on SARS-CoV-2 infection using definitive endoderm (DE) cells, the precursors of intestinal cell lineages. Msi1 expression in DE cells was silenced using lentiviral vectors encoding either Msi1-specific shRNA or scrambled shRNA, followed by infection with SARS-CoV-2. Viral replication in DE cells was assessed by immunofluorescence staining for dsRNA, a marker for active viral infection, and Sox17, a DE marker. Post-infection, ~1% of DE cells were positive for dsRNA, whereas Msi1-depleted cells showed a marked increase, with ~5% of the cells being dsRNA-positive. Importantly, Sox17 expression remained largely unaffected by Msi1 depletion, indicating that the depletion of Msi1 does not alter the DE cell population but specifically enhances viral infection (Fig. 5A). To further investigate the role of Msi1 in regulating SARS-CoV-2 infection under physiologically relevant conditions, we generated mid-hindgut organoids from induced pluripotent stem cells (iPSCs) and depleted Msi1 using shRNA (iPSCs; Supplementary Fig. S5A). Notably, Msi1-depleted organoids displayed a significant increase in nucleocapsid and dsRNA positivity compared to the control organoids (Fig. 5B and Supplementary Fig. S5B and C). Together, these findings identify Msi1 as a negative regulator of SARS-CoV-2 infection in intestinal models, highlighting its potential role in modulating viral susceptibility within the intestinal epithelium.

### Msi1 mediates SARS-CoV-2 inhibitory effects through repression of viral translation

**Figure 6.**
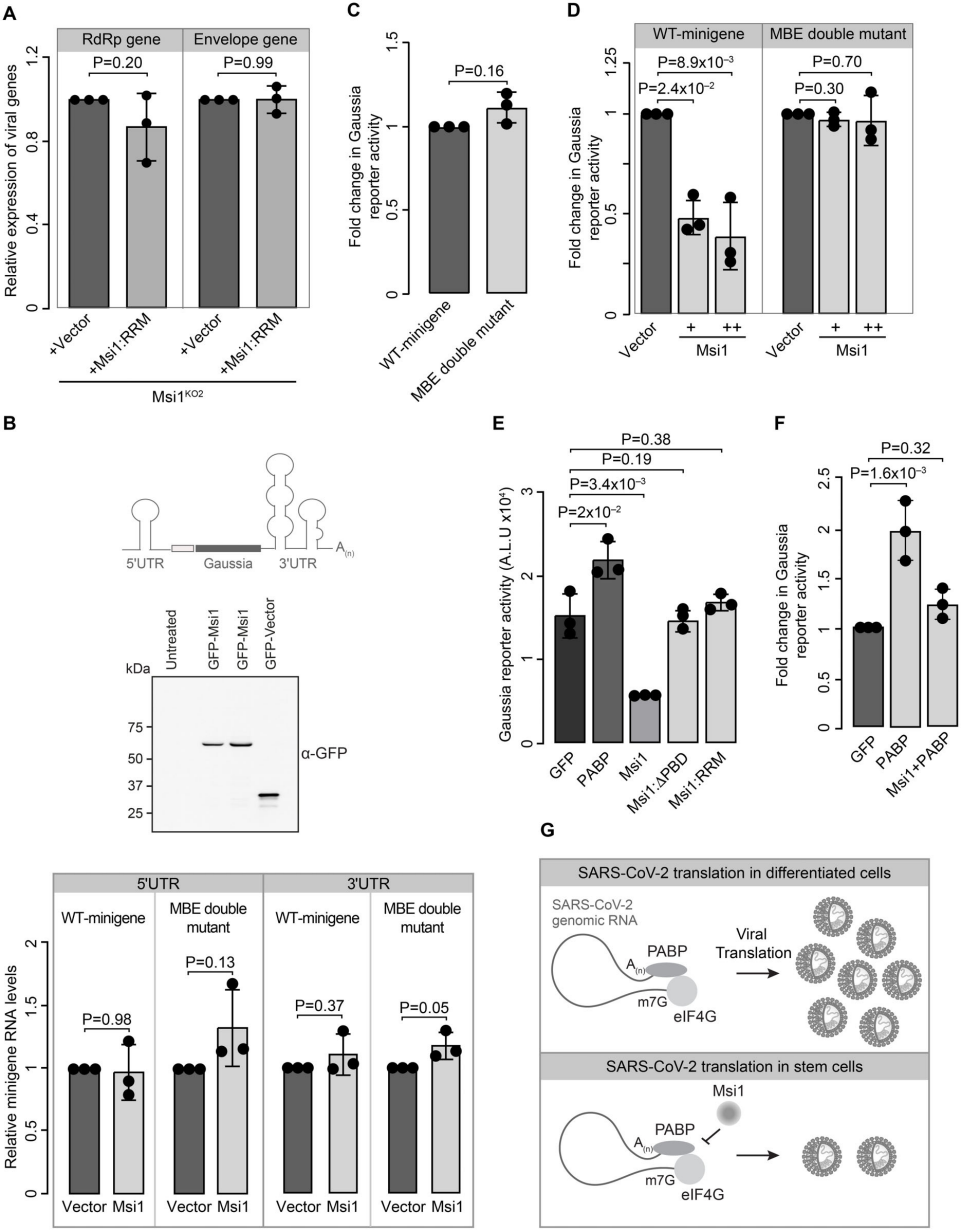
Msi1 represses SARS-CoV-2 translation via interaction with the viral 3′UTR. (**A**) Graph showing relative expression of RdRp and Envelope genes of SARS-CoV-2 in GFP- or GFP-RRM-expressed Msi1 KO cells. (**B**) Schematic representation of the minigene assay with the Gaussia reporter cloned between 3′UTR and 5′UTR of SARS-CoV-2 (top panel). Western blot below depicts the dose-dependent expression of GFP–Msi1 and GFP vector (middle panel). Graph depicts relative expression of wild-type and MBE-double mutant minigene transcripts assayed using primers against 5′UTR and 3′UTR in the presence of GFP vector and GFP–Msi1 (bottom panel). (**C**) Graph depicting fold change in the minigene Gaussia reporter activity with wild type or MBE double mutant 3′UTR from three independent experiments. (**D**) The graph represents fold change in the Gaussia reporter activity upon transfection of the minigene containing wild-type 3′UTR or the MBE double mutant transfected with vector or Msi1. (**E**) The graph represents fold change in Gaussia reporter activity upon transfection of the minigene containing the wild-type 3′UTR with GFP, PABP, Msi1, Msi1:ΔPBD (PABP non-binder), and Msi1:RRM only. (**F**) Quantification of change in WT minigene Gaussia reporter level upon transfection of GFP, PABP, and Msi1 + PABP. *P*-values were estimated using a two-tailed paired Student’s t-test. (**G**) Schematic representation of Msi1 binding to the 3′UTR and inhibiting viral translation in stem cells by possibly interfering with the PABP binding and translation of the viral proteins, resulting in a lesser number of viral particles.

Given that Msi1 possesses two conserved RRMs capable of interacting with RNA, we next examined whether these minimal RNA recognition motifs are sufficient to recapitulate the effects of the full-length protein on SARS-CoV-2. We performed a rescue experiment in Msi1 KO Caco-2 cells expressing RRMs (Msi1 1–190) along with GFP and GFP–Msi1, and examined changes in viral transcript levels. Surprisingly, the RRMs alone did not show a significant change in viral gene transcript levels, indicating that the intact Msi1 protein is required for the virus’s inhibition (Fig. 6A). Since Msi1 is a known translational repressor [48], we investigated whether its binding to the viral 3′UTR could impact viral translation. For this, we performed a luciferase reporter assay by transfecting the reporter mRNA tagged with the SARS-CoV-2 3′UTR, along with Msi1 (Supplementary Fig. S6A). Overexpression of Msi1 led to a 40%–50% reduction in the expression of the firefly luciferase/Renilla luciferase reporter from the wild-type 3′UTR. Interestingly, the trend towards dose dependency was not statistically significant (Supplementary Fig. S6B). This could be a result of aggregate formation at this concentration (Supplementary Fig. S6C). We also did not observe any significant difference in reporter expression in the MBE double mutant 3′UTR upon Msi1 transfection (Supplementary Fig. S6D). Given that the interactions between the 5′UTR and 3′UTR, along with their associated molecular interactions, are known to influence viral replication and translation [49], we performed a SARS-CoV-2 minigene reporter assay. In this construct, a Gaussia luciferase reporter is flanked by the 5′UTR and 3′UTR of SARS-CoV-2 and a segment of Nsp1. This *in vitro* transcribed construct closely mimics the viral replicon and can be used as a surrogate for viral translation [18]. We first examined and ensured that the RNA expression levels of the wild-type and double-mutant minigene transcripts were comparable upon mRNA transfection (Fig. 6B). We next analysed Gaussia reporter activity and found that the MBE double mutant does not affect reporter translation (Fig. 6C). Subsequently, we performed reporter assays employing the wild-type mini gene and the double mutant MBE containing the minigene transcript. While we observed a 50%–70% Msi1-driven decrease in Gaussia reporter activity in a dose-dependent manner for the minigene with the wild-type 3′UTR, the double mutant 3′UTR did not show any differences in the reporter activity (Fig. 6D). Taken together, these observations suggest that Msi1 binding to the M1 and M2 sites of SARS-CoV-2 3′UTR represses translation. Notably, SARS-CoV-2 RNAs are polyadenylated, which can influence their stability and translation [50]. For several coronaviruses, the interaction between the poly(A) tail and poly(A)-binding protein 1 (PABP1) has been shown to regulate viral translation [51, 52]. It is well established that Msi1 competes with PABP1, leading to translational repression [53]. We first observed that PABP alone could increase the Gaussia reporter level by ~2-fold compared to the vector. To assess whether removing the PABP-binding region in Msi1 affects translation, we generated a PABP-non-binding Msi1 (Msi1:ΔPBD) and performed a Gaussia reporter assay. Notably, while wild-type Msi1 repressed translation of the minigene, neither Msi1:RRM alone nor the Msi1 PABP non-binder could repress translation (Fig. 6E). This result was also mimicked by the 3′UTR reporter construct. (Supplementary Fig. S6E). To investigate whether Msi1 can inhibit the PABP-mediated translational upregulation of the SARS-CoV-2 minigene, we co-transfected equimolar amounts of PABP1 and Msi1 with the minigene. While we observed a 1.5- to 2.5-fold increase in translation upon PABP1 transfection, co-transfection of Msi1 reversed this upregulation, indicating that Msi1 could inhibit PABP1-mediated translation of SARS-CoV-2 (Fig. 6F). Taken together, our results indicate that both RNA binding and PABP-mediated binding can contribute to inhibitory effects of Msi1 on SARS-CoV-2 infection (Fig. 6G).

## Discussion

Similar to other RNA viruses, SARS-CoV-2 engages both canonical and non-canonical pathways of translation [54]. The 3′UTR of SARS-CoV-2 serves as a binding hub for host regulatory factors, as it contains several *cis-*acting elements essential for RNA synthesis and translation. Here, we show that (i) the SARS-CoV-2 3′UTR harbours two putative Msi1 binding elements (MBEs) in Stem Loop 1 (SL1) and hypervariable region (HVR); (ii) the weak *in vitro* binding observed at the M1 site, could imply that this region as a potential auxiliary or context-dependent Msi1 interaction site, with M2 serving as the dominant binding site within the SARS-CoV-2 3′UTR; (iii) the full-length Msi1 can repress SARS-CoV-2 translation; and (iv) this translational repression of SARS-CoV-2 might lead to reduced infectivity in Msi1-enriched stem cells. Previous studies have shown that Msi1 promotes Zika virus replication [3, 6], whereas in this study we demonstrate the translational repressor function of Msi1 in a viral context. Reporter assays using the 3′UTR and the mini-gene-based investigations show that Msi1 binds to 3′UTR and represses translation of SARS-CoV-2 genomic RNA in a dose-dependent manner. Notably, mere binding of Msi1 to the viral 3′UTR might not be sufficient to inhibit SARS-CoV-2 translation, as seen with the Msi1-RRM construct. Corroborating with this are our observations of the requirement of the PABP-binding region of Msi1, the presence of which is essential for translational repression.

Extensive characterization of RNA–protein interactions in SARS-CoV-2 across various human cell lines has been conducted recently [22, 55, 56]. While Msi1 has been identified as a potential interactor in Huh7 cells, it has not been detected as an interactor in other cell lines, such as HEK293 and Calu-3. The potential reasons for this discrepancy could be (i) cells do not express endogenous Msi1, as is the case in HEK293 [3]; (ii) downregulation of Msi1 protein upon SARS-CoV-2 infection, as shown here in both Calu3 and Huh7 cell lines; and (iii) expression of truncated forms of Msi1, for instance, the Msi1 expressed in Calu3 lacks the C-terminus, which might influence the viral RNA-host protein interaction (Supplementary Fig. S7A).

Msi1 is known to be a stem cell marker [39]. Indeed, single-cell gene expression analysis of intestinal cells revealed that Msi1 is expressed in transient amplifying (TA) cells and stem cells. Importantly, we demonstrate that DE cells, which express high levels of Msi1, are less permissive to SARS-CoV-2 infection, despite expressing ACE2. Depletion of Msi1 in DE cells shows increased infection, establishing the antiviral role of Msi1. On the other hand, the differentiated enterocytes that are readily infected by SARS-CoV-2 show negligible expression of Msi1 (Supplementary Fig. S7B). Akin to our observations in intestinal stem cells, neural progenitors in cerebral organoids express high levels of Msi1 (Supplementary Fig. S7C). The findings presented here may partially explain why Msi1-enriched neural progenitors are less susceptible to SARS-CoV-2 infection [9].

To capture the expression alterations of host genes during infection, we analysed publicly available single-cell RNA-seq data of intestinal organoids infected with SARS-CoV-2 and found that Msi1 transcript significantly goes down at the 12 h time point (Supplementary Fig. S7D). Integrating these observations with our findings of Msi1-mediated repression of SARS-CoV-2, it is tempting to speculate that the virus most likely downregulates Msi1 during the onset of its gRNA translation. However, when the innate immune response sets in at 24 h time point, enhanced expression of Msi1 is observed. It is now well known that different stem cells have cell type-specific groups of interferon-stimulated genes, which makes the stem cells resistant to viral infection compared to their differentiated progeny [57–59]. In addition to directly binding and repressing viral translation, Msi1 can also activate specific modules, such as protein kinase R, which are activated by dsRNA and can restrict viral translation by inducing a shutdown [60–62]. Thus, an interplay between the innate immune response and RBPs, such as Msi1, could play a vital role in viral containment and warrants further exploration.

## Supplementary Material

gkag271_Supplemental_File

## Data Availability

An in-house Python script has been developed, encapsulating the methodology provided in the Perl utility plfoldz.pl, available at https://github.com/mtw/plfoldz and https://doi.org/10.6084/m9.figshare.31672210. All data are available in the main text or the supplementary materials.

## Notes

Present address: PopVax Pvt Limited, Hyderabad, Telangana 500039, India

Present address: Department of Biological Sciences and Mechanobiology Institute, National University of Singapore, Singapore 117411, Singapore
