# Correction to ‘RNA binding protein Musashi1 interacts with the viral genomic RNA and restricts SARS-CoV-2 infection by repressing translation’

**DOI:** 10.1093/nar/gkag358

**Published:** 2026-04-17

This is a correction to: Sourav Ganguli, Divya Gupta, Rajashekar Varma Kadumuri, Dixit Tandel, Rajashree Ramaswamy, Aswathy G Krishnan, Deena T David, Soumya Bunk, Sreenivas Chavali, Krishnan Harinivas Harshan, Pavithra L Chavali, RNA binding protein Musashi1 interacts with the viral genomic RNA and restricts SARS-CoV-2 infection by repressing translation, *Nucleic Acids Research*, Volume 54, Issue 6, 13 April 2026, gkag271, https://doi.org/10.1093/nar/gkag271

The following statement was omitted at the original publication but is now added to the end of the **Funding** Section of the paper: “Funding to pay the Open Access publication charges for this article was provided by ONOS, an Initiative of the Government of India.”

