# Supplementary material for: RNA binding protein Musashi1 interacts with the viral genomic RNA and restricts SARS-CoV-2 infection by repressing translation
Source: Nucleic Acids Res. 2026 Mar 31;54(6):gkag271. doi: 10.1093/nar/gkag271 (PMC13036491; doi:10.1093/nar/gkag271)
Supplement: gkag271_Supplemental_File [file gkag271_supplemental_file.pdf]

Supplementary Information for

RNA binding protein Musashi1 interacts with the viral genomic RNA and restricts SARS-CoV-2 infection by repressing translation

Sourav Ganguli[1,2,3], Divya Gupta[1] , Rajashekar Varma Kadumuri[3], Dixit Tandel[1,2#] Rajashree Ramaswamy[1,4#] , Aswathy G Krishnan[3], , Deena T David[1,2], Soumya Bunk[1,5], Sreenivas Chavali[3], Krishnan Harinivas Harshan[1,2], Pavithra L. Chavali[1,2,3*]

[1]CSIR-Centre for Cellular and Molecular Biology, Hyderabad- 500007, Telangana, India

[2]Academy of Scientific and Innovative Research (AcSIR), Ghaziabad- 201002, Uttar Pradesh, India

[3]Department of Biology, Indian Institute of Science Education and Research (IISER) Tirupati, Yerpedu, Tirupati- 517619, Andhra Pradesh, India

*Correspondence: pavithrachavali@iisertirupati.ac.in

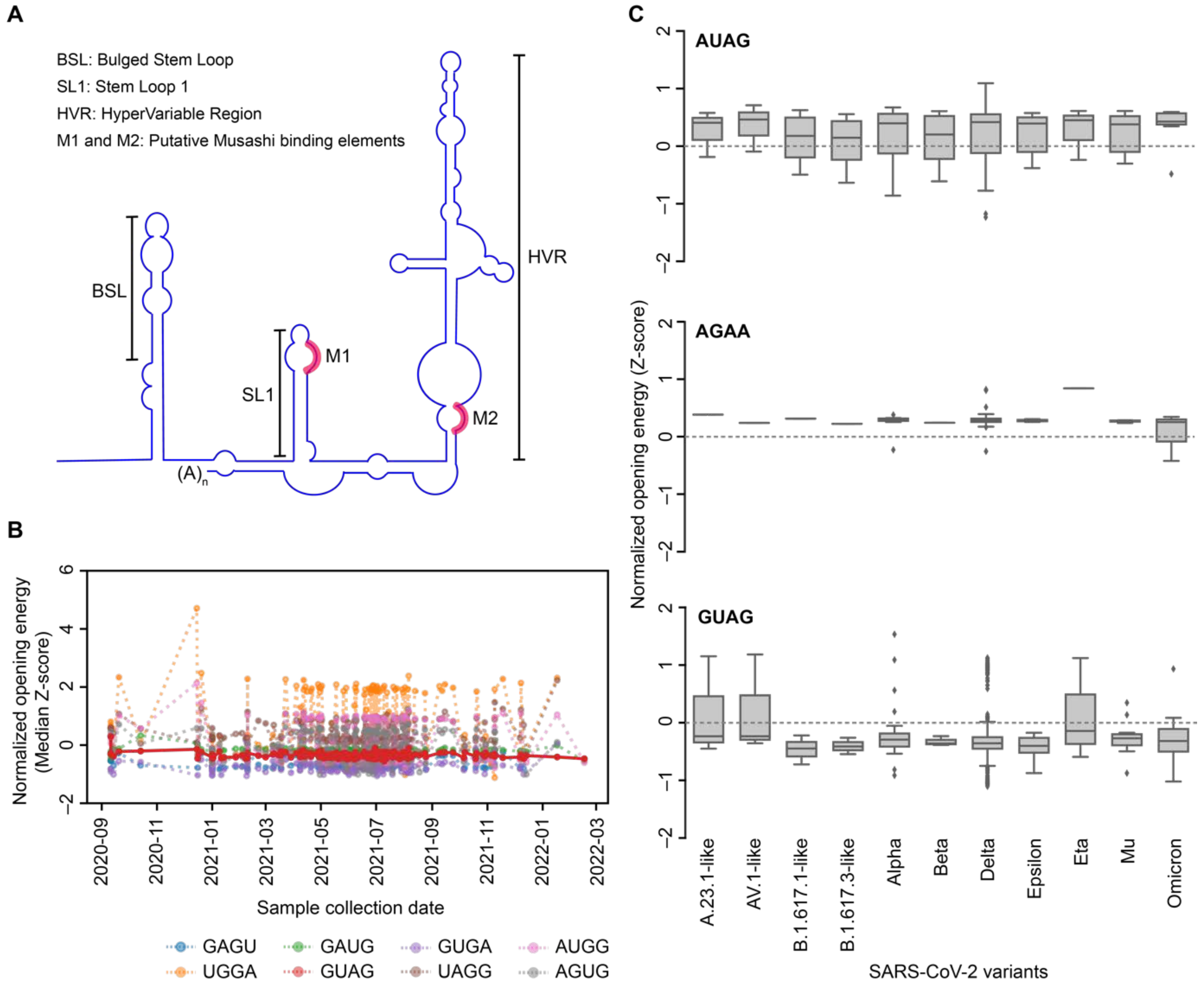

Supplementary Figure 1. Musashi Binding Elements in the 3'UTR of the SARS-CoV-2 genome. (A) The 3'UTR of SARS-CoV-2 forms a distinct secondary structure with two prominent MBEs - M1 and M2 in the stem-loop1 (SL1) and hyper variable region (HVR), respectively. **(B)** Graph showing the Z-scores of the normalised opening energy of different motifs in SARS-CoV-2 variants, with randomized GUAG consensus, collected over different time points. **(C)** Boxplot of distributions of the Z-scores of the normalised opening energy of the different consensus MBEs (AUAG, upper panel; AGAA, middle panel and GUAG, lower panel) among different SARS-CoV-2 variants.

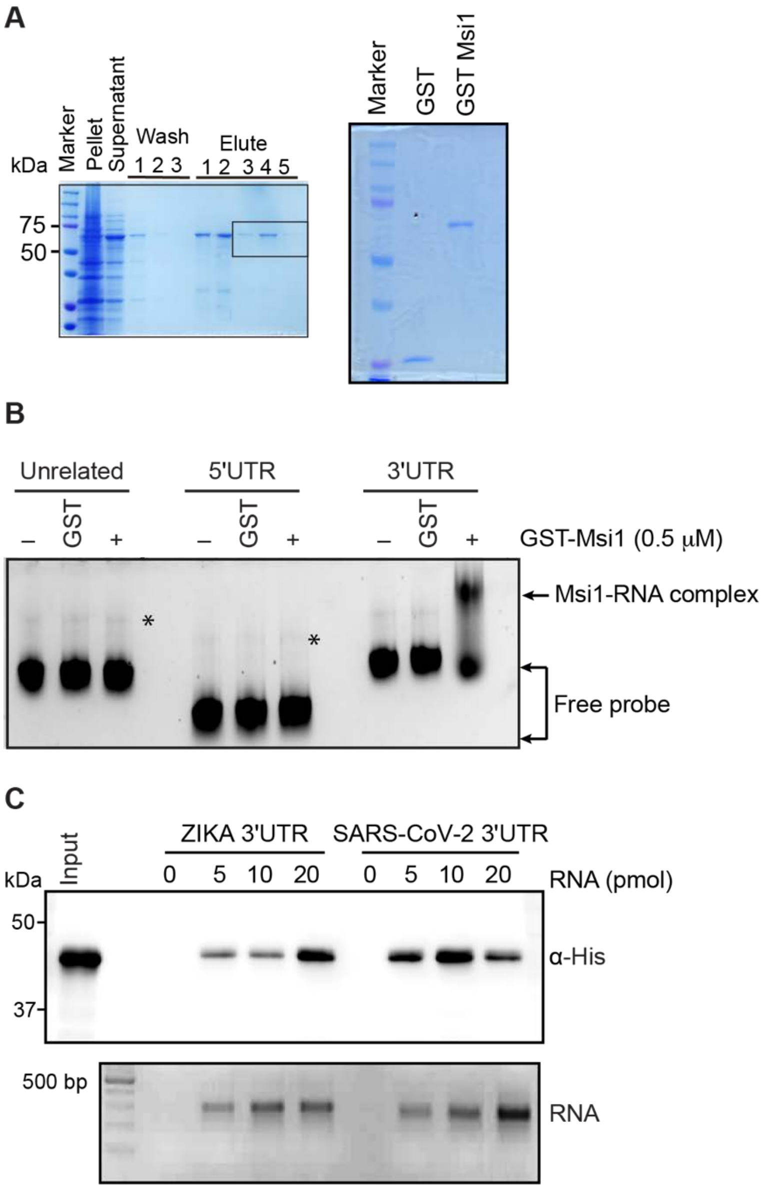

Supplementary Figure 2. Recombinant Msi1 protein binds to 3'UTR of SARS-CoV-2. (A) Coomassie gel depicting GST-Msi1 purification. The last three elutes were collected and concentrated using a MWCO 30 column. GST and the GST Msi1 proteins used for binding studies as depicted in the gel on the side. **(B)** RNA EMSA depicting specific binding of Msi1 to 3'UTR probe, while the 5'UTR and the non-specific RNA of the same size did not show binding. **(C)** RNA pull-down assays performed with the 3' UTRs of SARS-CoV-2 and Zika virus. In vitro transcribed biotinylated 3' UTR RNA from SARS-CoV-2 and Zika virus were incubated with His or His-Msi1 and RNA-protein complexes were captured on streptavidin beads.

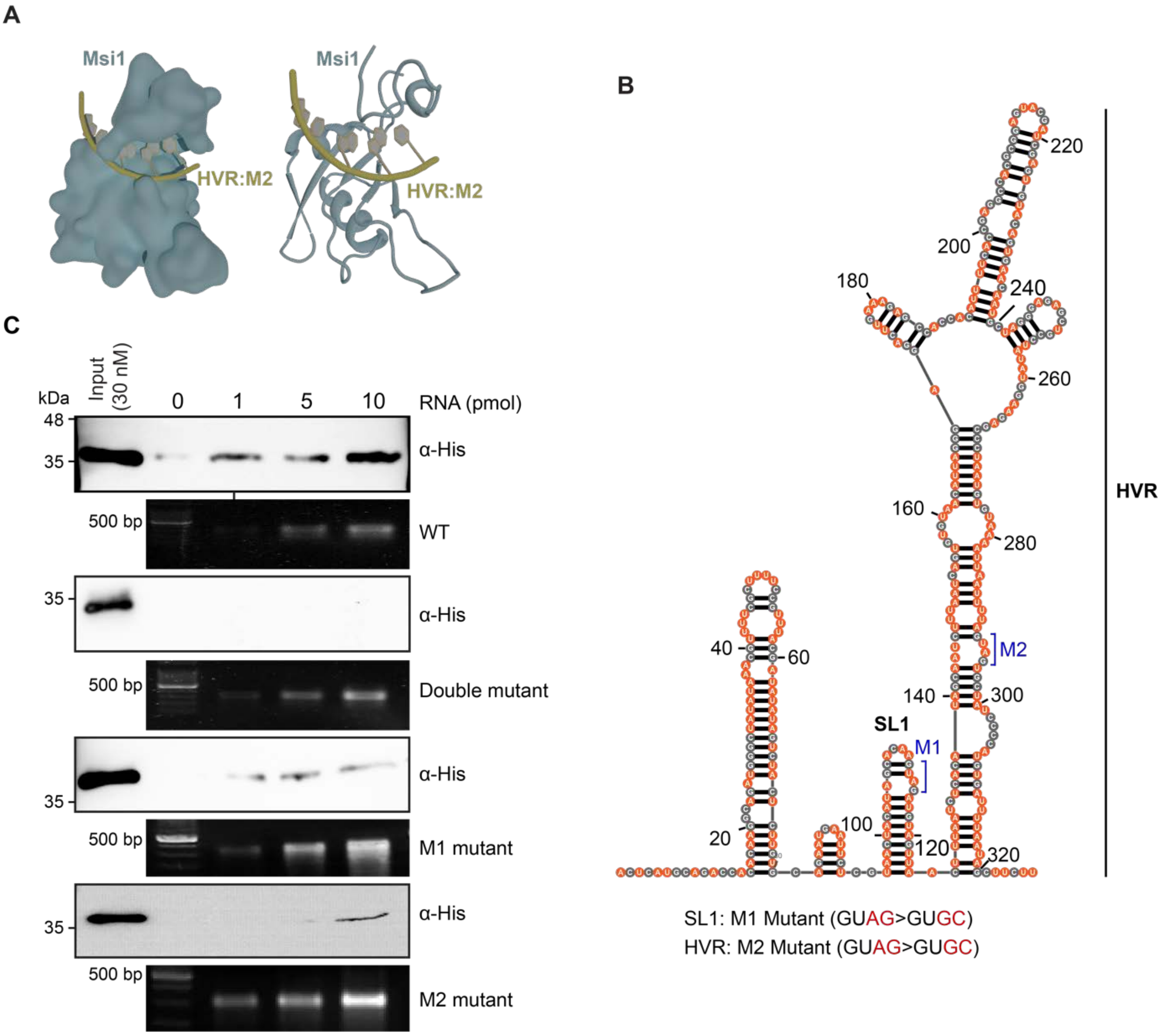

Supplementary Figure 3. Msi1 directly interacts with SARS-CoV-2 3'UTR via MBEs. (A) Multifaceted visualization of Msi1 (PDBID:5X3Z) and SARS-COV-2 M2 RNA motif interaction. The left panel shows the surface representation of the Msi1 in complex with viral motif M2, highlighting the detailed interaction surface, including key binding sites and molecular contours. The right panel presents a cartoon representation of the same complex, emphasizing the overall structural arrangement and folding patterns of the Msi1 and the viral motif M2. **(B)**The 3'UTR of SARS-CoV-2 forms a distinct secondary structure with two prominent MBEs- M1 and M2. **(C)** RNA pull down of recombinant His-Msi1 protein with in vitro transcribed wildtype 3'UTR of SARS-CoV-2 and individual mutations in M1, M2, and the double mutant, followed by immunoblot analysis probed with His-tag antibody. RNA input for the respective RNA pulldown has been shown below each immunoblot.

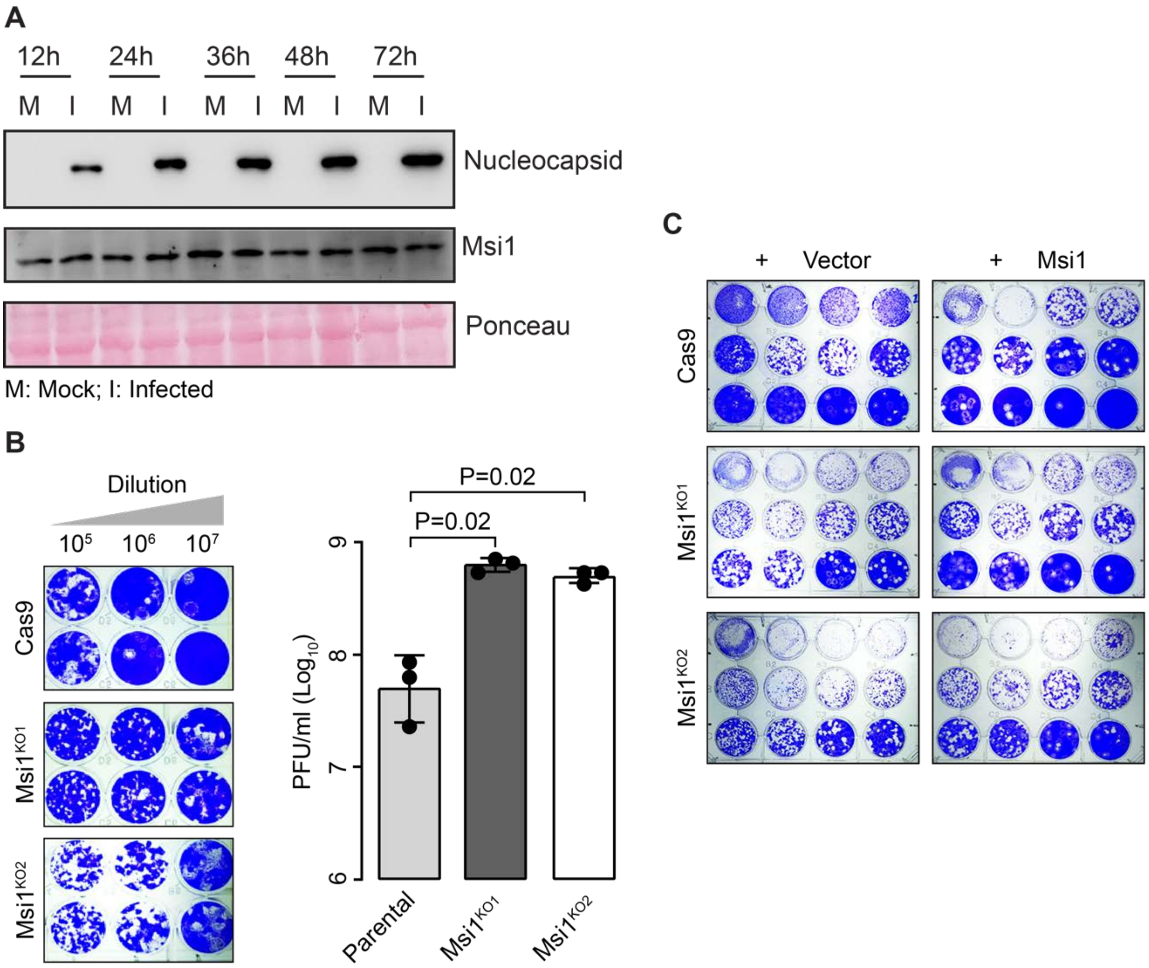

Supplementary Figure 4. Msi1 affects viral replication and infectivity (A) Immunoblot analysis of Nucleocapsid and Msi1 protein in Mock (M) and SARS-CoV-2 infected (I) samples at indicated timepoints **(B)** Panel on the left shows a representative image of Plaques. The graph on the right shows the relative fold change in infectious viral titers of SARS-CoV-2 in Parental or Msi1 KO samples compared to those treated with vehicle, represented as fold change in PFU/mL. **(C)** Plate images depict the difference in plaque formation between wild-type Caco-2 cells and Msi1 KOs with transgene expression of Msi1 or an empty vector.

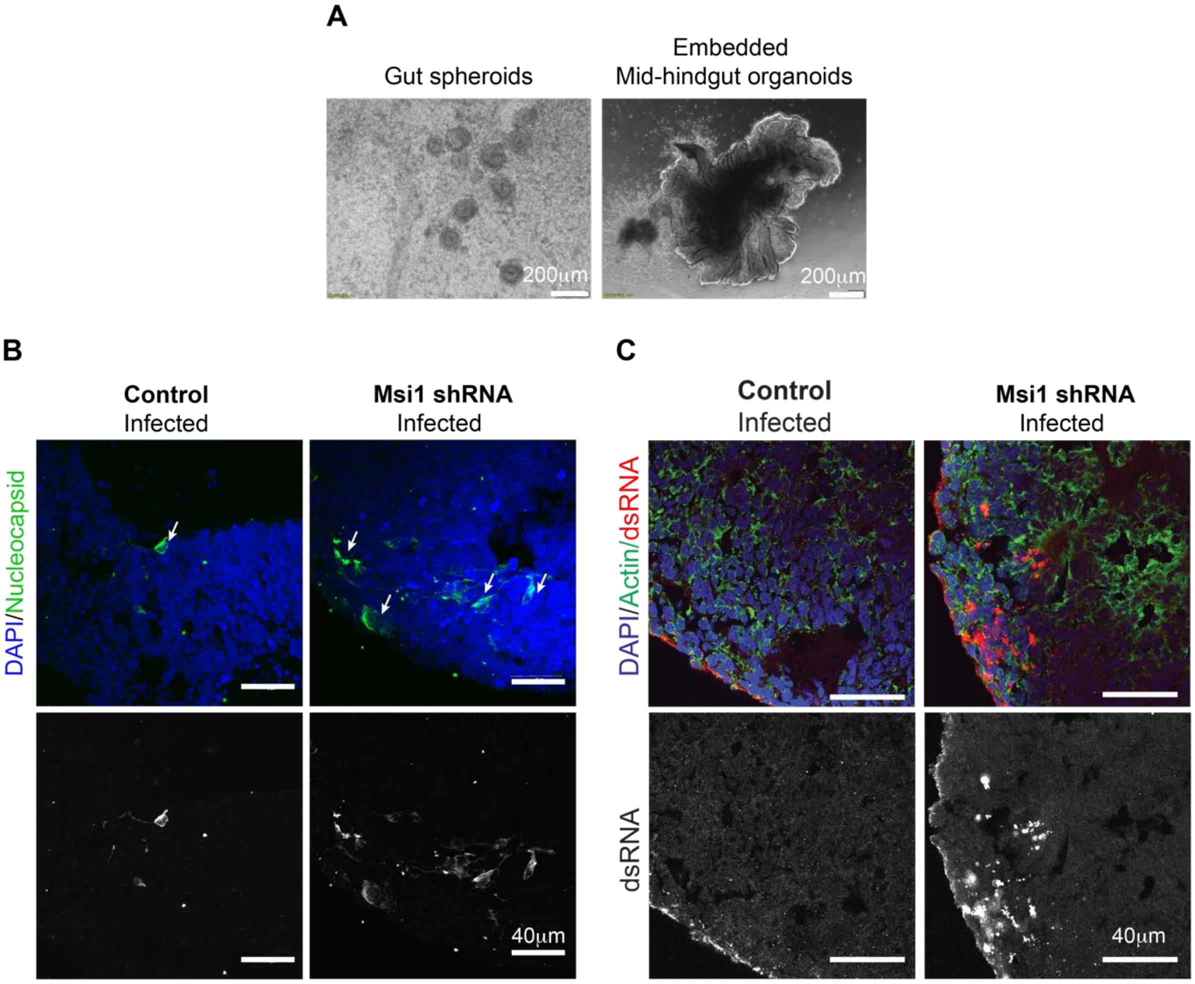

Supplementary Figure 5. Msi1 depletion promotes viral replication in 3D cultures. (A) Representative images of gut spheroids and mid-hindgut organoids used for SARS-CoV-2 infection. Immunostaining of **(B)** Nucleocapsid (green) and **(C)** dsRNA (red) and Actin (green) in organoids treated with control or Msi1 ShRNA after 48 h. DAPI is stained in blue for both.

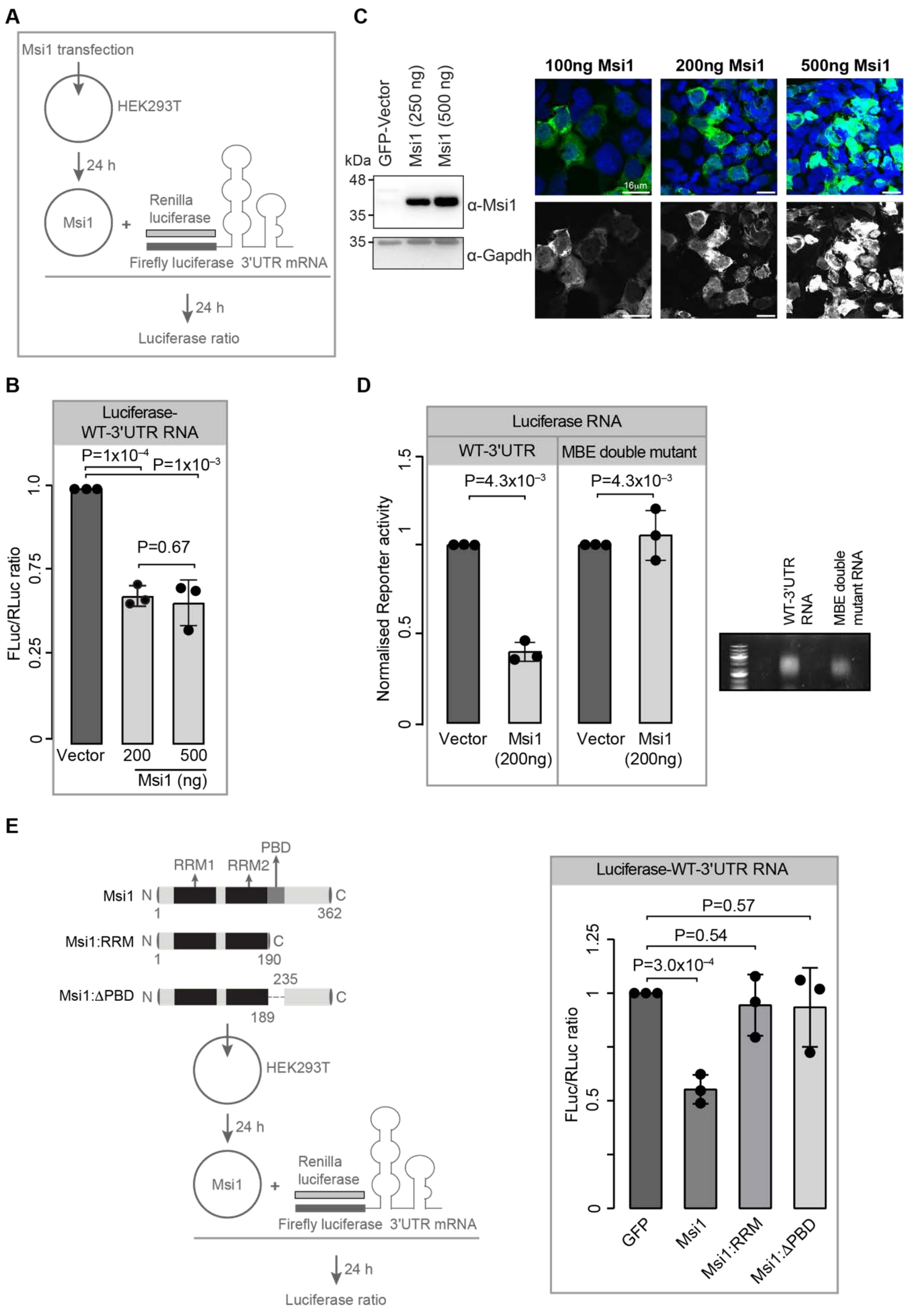
A
Msi1 transfection
HEK293T
24 h
Msi1
Renilla luciferase
Firefly luciferase 3'UTR mRNA
Luciferase ratio
B
Luciferase-WT-3'UTR RNA
P=1x10⁻⁴
P=1x10⁻³
P=0.67
FLuc/RLuc ratio
Vector
200
500
Msi1 (ng)
C
GFP-Vector
Msi1 (250 ng)
Msi1 (500 ng)
kDa
α-Msi1
α-Gapdh
100ng Msi1
200ng Msi1
500ng Msi1
16µm
D
Luciferase RNA
WT-3'UTR
MBE double mutant
P=4.3x10⁻³
Normalised Reporter activity
Vector
Msi1 (200ng)
WT-3'UTR RNA
MBE double mutant RNA
E
RRM1
RRM2
PBD
Msi1
Msi1:RRM
Msi1:ΔPBD
HEK293T
24 h
Renilla luciferase
Firefly luciferase 3'UTR mRNA
Luciferase ratio
Luciferase-WT-3'UTR RNA
P=0.57
P=0.54
P=3.0x10⁻⁴
FLuc/RLuc ratio
GFP
Msi1
Msi1:RRM
Msi1:ΔPBD

Supplementary Figure 6. Msi1 represses viral translation. **(A)** Schematic of the dual-luciferase reporter assay using the SARS-CoV-2 WT 3'UTR. HEK293T cells were transfected with Msi1 followed by co-transfection of firefly luciferase reporter RNA containing the viral 3'UTR and Renilla luciferase as a normalization control. Luciferase activity was measured after 24h. **(B)** Quantification of firefly luciferase activity normalized to Renilla luciferase in the presence or absence of Msi1 using the SARS-CoV-2 3'UTR reporter. **(C)** Immunoblot showing dose-dependent expression of Msi1 in HEK293T cells with GAPDH as a loading control. Representative immunofluorescence images on the right show increased Msi1 expression and aggregate formation upon higher plasmid transfection. **(D)** Dual-luciferase reporter assay comparing repression mediated by the WT or MBE double mutant SARS-CoV-2 3'UTR in the presence of Msi1. Agarose gel image on the right confirms the integrity of the *in vitro* transcribed (IVT) reporter RNA used for transfection. **(E)** Schematic of Msi1 truncation constructs used in the reporter assay. The graph on the right shows normalized luciferase activity following co-expression of WT 3'UTR reporter RNA with full-length or truncated Msi1 constructs. Data are normalized to GFP vector control.

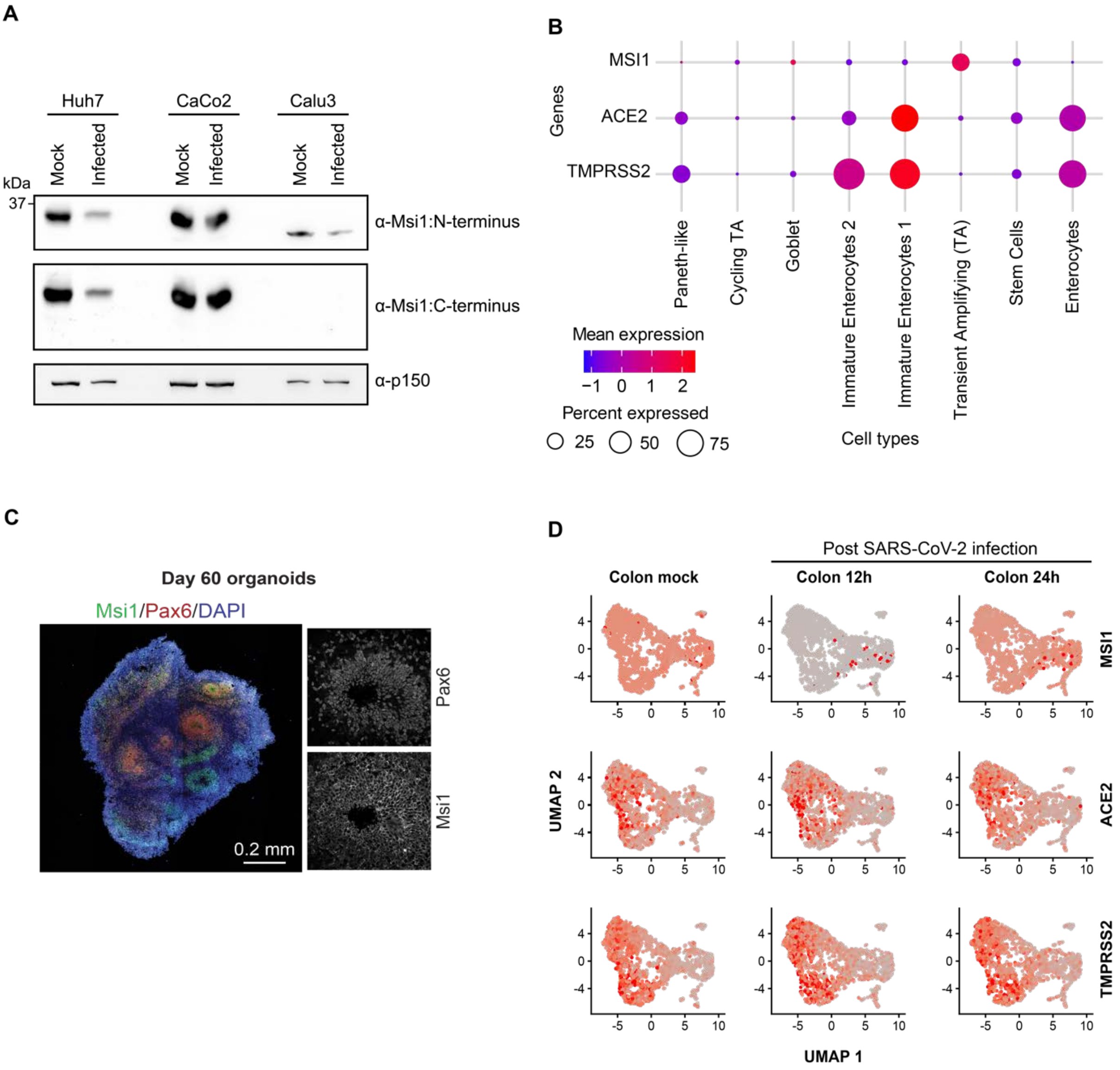

Supplementary Figure 7. Msi1 expression upon SARS-CoV-2 infection. (A) Western blot analysis of Msi1 in Huh7, Caco-2 and Calu-3 cell lines after mock or SARS-CoV-2 infection. P150 was used as a loading control. **(B)** Bubble plot, depicting expression levels of MSI1, ACE2 and TMPRSS2 across cell clusters in the intestine from publicly available dataset (1). The color of each bubble represents mean level of expression of the indicated gene whereas the relative size of the bubble is indicative of the percentage of cells expressing the gene. **(C)** Confocal image analysis of a D60 cerebral brain organoid expressing Msi1(green) and Pax6 (red) or Msi2 (green) and Pax6 (red). DAPI (blue) stains nuclei. **(D)** Uniform manifold approximation and projection (UMAP) embedding of the scRNAseq data of infected colon organoids at 12 and 24 h post-infection, colored by the corrected targeted normalized expression of SARS-CoV-2.

Supplementary References

1. Triana, S., Metz-Zumaran, C., Ramirez, C., Kee, C., Doldan, P., Shahraz, M., Schraivogel, D., Gschwind, A.R., Sharma, A.K., Steinmetz, L.M. *et al.* (2021) Single-cell analyses reveal SARS-CoV-2 interference with intrinsic immune response in the human gut. *Mol Syst Biol*, **17**, e10232.
